# Machine learning analysis of surface electromyography for quantifying neuromuscular compensatory profiles and associated functional disability in neck pain

**DOI:** 10.3389/fbioe.2026.1804580

**Published:** 2026-08-11

**Authors:** Changxiao Han, Bochen Peng, Hongtao Li, Jiacheng Zheng, Xu Wang, Zhiwen Li, Jiali Chen, Guangyi Yang, Guangwei Liu, Liguo Zhu, Minshan Feng

**Affiliations:** 1 Wangjing Hospital, China Academy of Chinese Medical Sciences, Beijing, China; 2 Beijing University of Chinese Medicine, Beijing, China

**Keywords:** compensatory strategies, functional assessment, machine learning, neck pain, neuromuscular dysfunction, SHAP analysis, surface electromyography

## Abstract

**Background:**

Neck pain presents significant clinical heterogeneity, while current assessment methods lack systematic approaches for quantifying muscle function. This study established a multi-dimensional surface electromyography (sEMG) framework to quantify the predictive value of muscle function features for clinical outcomes and identify neuromuscular compensatory states.

**Methods:**

Eighty-nine participants were enrolled, including 69 chronic neck pain patients and 20 age-, sex-, and BMI-matched healthy volunteers. Surface EMG signals from six bilateral cervical muscles were recorded during functional postural control tasks in four directions. Following preprocessing and quality control, seven features spanning coordination (co-contraction index [CCI], deep-to-superficial ratio [DSR], asymmetry index [ASI]), control (sample entropy [SampEn], coefficient of variation trend [CoV_trend]), and fatigue (initial fatigue rate [IFR], median frequency slope [MDF_Slope]) dimensions were extracted. Six regression models (multiple linear regression, Ridge, Lasso, ElasticNet, Random Forest, and XGBoost) were compared using Repeated K-Fold cross-validation (5 folds × 10 repeats), with SHapley Additive exPlanations (SHAP) quantifying feature contributions. K-means clustering with multi-index validation identified distinct neuromuscular states.

**Results:**

Data quality reached 95.3% (263/276 segments). Six of seven EMG features significantly discriminated between healthy controls and patients (p < 0.05), with five showing large effect sizes (|d| > 0.8). Comparative model evaluation revealed XGBoost as the superior predictor for NDI scores (CV *R* ^2^ = 0.449 ± 0.182, MAE = 4.66 ± 0.74), outperforming all linear models by 13%–16%; a permutation test confirmed statistical significance (p = 0.001). SHAP analysis identified CCI (mean |SHAP|: 3.68), CoV_trend (1.74), and MDF_Slope (1.56) as primary NDI predictors. Four features exhibited significant severity gradients: CCI progressively increased from mild (0.57 ± 0.06) to severe groups (0.73 ± 0.10, p < 0.001); DSR was significantly higher in mild versus moderate patients (1.73 vs. 1.37, p = 0.033); CoV_trend decreased with severity (p = 0.013); MDF_Slope showed progressive changes (p < 0.001). Multi-index clustering validation (Silhouette, Calinski-Harabasz Index, Davies-Bouldin Index, Gap Statistic) supported K = 2 as the optimal cluster number. Two candidate neuromuscular states were identified: Asymmetric Compensation (63.8%, high ASI: 26.7%, preserved DSR: 1.744, low CCI: 0.635) and Global Stiffening (36.2%, high CCI: 0.771, low DSR: 1.077, steep MDF_Slope: −0.408), consistent with established models of pain-motor reorganization. Cluster membership was associated with disease severity (χ^2^ = 6.06, p = 0.048). EMG-NDI associations were independent of demographic confounders (partial correlations virtually identical to zero-order values after controlling for age, sex, and BMI; maximum change <0.1%).

**Conclusion:**

This interpretable multi-dimensional sEMG framework quantifies muscle function features’ predictive value for functional disability. Severity-related neuromuscular changes may enable disease progression monitoring, while the identification of candidate compensatory states provides preliminary stratification criteria for personalized rehabilitation pending prospective validation.

## 1 Introduction

Neck pain represents a major global health burden, with a point prevalence of 4.9% and ranking fourth in years lived with disability according to the Global Burden of Disease 2010 Study (Hoy et al., 2014; GBD, 2021 Neck Pain Collaborators et al., 2024). Approximately 30%–50% of adults experience neck pain during their lifetime, resulting in substantial functional limitations, reduced quality of life, and significant socioeconomic costs (Cohen, 2015; Fejer et al., 2006). Despite advances in diagnostic techniques and therapeutic interventions, the clinical management of neck pain remains challenging due to its complex, multifactorial etiology and heterogeneous clinical presentations (Haldeman et al., 2010).

Cervical muscle function, encompassing muscle coordination, motor control quality, and fatigue resistance, plays a critical role in neck pain patients’ symptom severity and functional disability (Falla et al., 2004a; Schomacher and Falla, 2013; Johnston et al., 2008). Abnormal co-contraction patterns, deep muscle weakness, asymmetric muscle activation, and impaired functional endurance have been closely associated with the development and progression of neck pain (Falla et al., 2004b; Jiménez-Grande et al., 2021; Cheng et al., 2010). However, current clinical assessment methods, including imaging modalities and physical examination, are limited in their ability to objectively quantify cervical muscle functional status and neuromuscular control quality. Surface electromyography (sEMG) provides a non-invasive approach for objective assessment of muscle function.

Surface EMG enables real-time, simultaneous multi-muscle recordings (Merletti and Farina, 2016) and quantification of muscle coordination, motor control quality, and neuromuscular fatigue (Farina et al., 1985; De Luca, 1997; Cifrek et al., 2009), with demonstrated utility in spinal disorder assessment (Falla et al., 2014; Jiménez-Grande et al., 2022; Muceli et al., 2014). Nevertheless, prior sEMG research in neck pain has three notable limitations. First, most studies have examined isolated muscles or single features during specific clinical tests—such as deep cervical flexor activation during the craniocervical flexion test (Falla et al., 2004b) or cervical motor strategy reorganization (Falla and Farina, 2008)—rather than integrating multi-muscle, multi-feature profiles during functional tasks. Second, machine learning applications to cervical or spinal EMG data have primarily pursued binary classification (patient vs. healthy (Jiménez-Grande et al., 2021); pain subtypes (Chen and Guestrin, 2016)) rather than continuous feature–outcome quantification with interpretable methods such as SHAP (Molnar, 2022). Third, no study has unified coordination, motor control, and fatigue dimensions within a single framework, nor quantified each dimension’s contribution to functional disability on a continuous scale.

Addressing these limitations requires a systematic approach that simultaneously captures complementary aspects of neuromuscular function and links them transparently to clinical outcomes. Furthermore, criteria for patient stratification based on muscle function profiles have not been established, limiting the development of targeted rehabilitation strategies. Advancing these aspects is essential for optimizing clinical management of neck pain.

The present study aimed to: (1) establish a multi-dimensional EMG feature framework based on functional postural control tasks, encompassing coordination (co-contraction, muscle balance, asymmetry), control (complexity, stability), and fatigue (early fatigue, sustained fatigue) domains; (2) quantify the predictive value of individual features for functional disability (Neck Disability Index, NDI) and pain intensity (Visual Analog Scale, VAS) using machine learning approaches; and (3) explore distinct neuromuscular compensatory states in neck pain patients based on muscle function feature profiles through unsupervised clustering analysis.

## 2 Methods

### 2.1 Participants

A total of 89 participants were enrolled from Chinese Academy of Chinese Medical Sciences Wangjing Hospital between 2025/01/01 and 2025/06/30, comprising 69 patients diagnosed with cervical spondylosis and 20 healthy volunteers. Patient inclusion criteria were: (1) clinical diagnosis of cervical spondylosis according to established criteria; (2) age 18–65 years; (3) ability to perform cervical range of motion tasks. Exclusion criteria included: (1) cervical spine surgery history; (2) acute cervical trauma; (3) neurological disorders affecting motor control; (4) contraindications to surface EMG recording. Healthy volunteers were recruited from hospital staff and community volunteers with no history of neck pain, cervical spine disorders, or neurological conditions within the preceding 12 months. Healthy volunteers were matched to the patient group for age, sex, and BMI (all p > 0.05). All participants provided written informed consent. This study was approved by the Ethics Committee of Chinese Academy of Chinese Medical Sciences Wangjing Hospital (WJEC-KT-2024–025-P002). Clinical severity was assessed using the Neck Disability Index (NDI, 0–50). Participants were classified into three severity groups: Mild (NDI: 0–14), Moderate (NDI: 15–24), and Severe (NDI ≥25).

### 2.2 EMG data acquisition

Surface EMG signals were recorded from six bilateral cervical muscles using [Equipment Model: Pico, Manufacturer: cometa]. Electrode placement followed standardized anatomical landmarks: bilateral semispinalis capitis (L.SC, R.SC), upper trapezius (L.UT, R.UT), and sternocleidomastoid (L.SCM, R.SCM). All electrode placements were performed by two trained operators following identical anatomical landmark protocols and standardized skin preparation procedures. Ag/AgCl electrodes (diameter: 10 mm, inter-electrode distance: 25 mm) were positioned after skin preparation (alcohol cleaning, impedance <5 kΩ). A reference electrode was placed on the C7 spinous process. Signals were sampled at 2000 Hz with 16-bit resolution. The acquisition system supports a common-mode rejection ratio (CMRR) > 110 dB, input impedance > 10^15 Ω, signal bandwidth of 10–500 Hz, 16-bit analog-to-digital resolution, and a 2000 Hz per-channel sampling rate. To safeguard signal quality, electrode–skin impedance was kept below 5 kΩ for every channel after standardized skin preparation (abrasion and 70% isopropyl alcohol). A retrospective robustness analysis of the acquisition architecture—covering signal-to-noise ratio, integrity, electromagnetic interference (EMI) attenuation, inter-channel cross-talk, signal stationarity, channel reliability and AI-performance sensitivity—was performed following the framework principles outlined in prior work on embedded biomedical acquisition validation (Vincenzi et al., 2026a) (Supplementary Figure S7; Supplementary Table S15).

**FIGURE 1:**
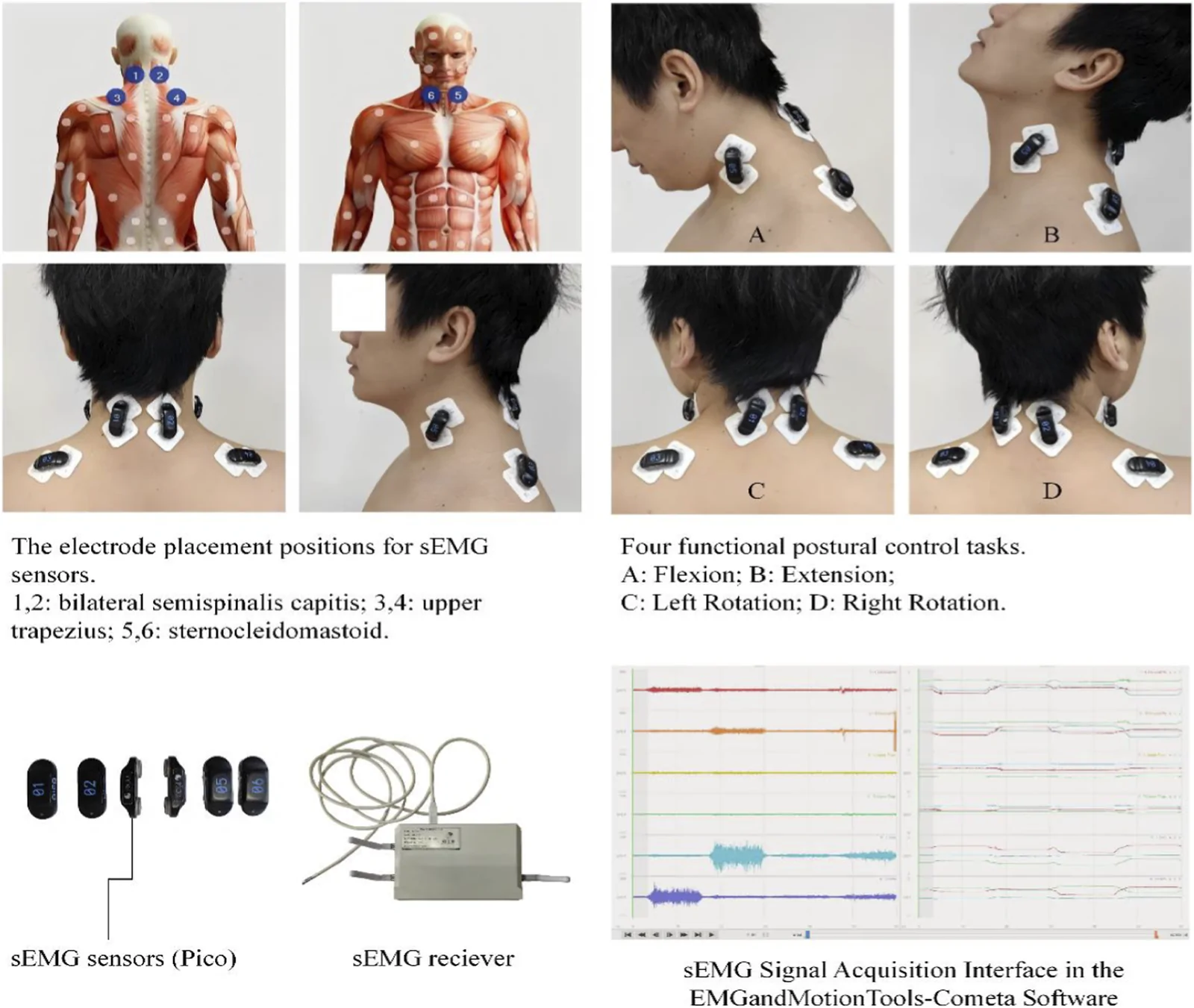
Surface electromyography (sEMG) experimental setup and data acquisition protocol.

Participants sat in a standardized position with feet flat on the ground and knees and hips flexed at 90°. Four functional postural control tasks were recorded: flexion, extension, left rotation, and right rotation. For each task, participants actively moved to their maximum comfortable angle and maintained the position for 30 s under therapist supervision (Figure 1). From each 30-s recording, the middle 10-s segment was first extracted to exclude unstable signals during task initiation and termination. Subsequently, a sliding window algorithm selected the most stable 5-s segment based on composite criteria including RMS envelope variance minimization and acceleration stability (Supplementary Figure S2). The 5-s window provides 10,000 data points at 2000 Hz sampling, sufficient for reliable estimation of SampEn (requiring N > 200 for m = 2) (Pincus, 1991) and frequency-domain features including MDF slope regression (De Luca, 1997; Cifrek et al., 2009).

### 2.3 Signal preprocessing and quality control

Raw EMG signals were preprocessed using custom Python scripts (Python 3.8, NumPy 1.21.0, SciPy 1.7.0): 50 Hz notch filter, 20–450 Hz 4th-order Butterworth bandpass filter, and full-wave rectification. Signal envelopes were extracted using a 50 ms moving average window. Power spectral density was calculated using 256-point FFT with 512 ms windows (50% overlap). Data quality was assessed using a seven-dimensional composite scoring system incorporating signal-to-noise ratio (weight 30%), signal saturation (15%), crest factor (15%), RMS stability (15%), spectral concentration (10%), zero-crossing rate (10%), and postural stability (5%). Composite scores ranged from 0 to 10, with a threshold ≥6.0 defining acceptable samples (Supplementary Figures S1–S2; Supplementary Table S1). The stability of this threshold was evaluated through patient-level split-half validation (1,000 random splits) comparing pass rates between halves, and bootstrap resampling (n = 2,000) to estimate the 95% confidence interval of the overall pass rate.

### 2.4 Feature extraction

Feature extraction was performed in two tiers. First, basic physiological features were extracted from each muscle channel of the 5-s stable segment (Supplementary Table S2): time-domain features (RMS, MAV, WL) and frequency-domain features (MDF, MNF). Based on these baseline features, seven composite functional indicators spanning three clinical dimensions were calculated: coordination (Co-contraction Index [CCI], Deep-to-Superficial Ratio [DSR], Asymmetry Index [ASI]), control (Sample Entropy [SampEn], Coefficient of Variation Trend [CoV_trend]), and fatigue (Initial Fatigue Rate [IFR], Median Frequency Slope [MDF_Slope]). Detailed definitions, mathematical formulations, and parameter specifications for each feature are provided in Table 1.

**TABLE 1:** Definitions and mathematical formulations of seven core EMG features.

Feature	Dimension	Formula	Parameters
CCI	Coordination	CCI = 2 × ∫ min (EMG_A, EMG_B)dt/∫(EMG_A + EMG_B)dt	EMG_A, EMG_B: RMS envelopes of antagonist muscle pairs (SCM vs. SC) during each movement direction
DSR	Coordination	DSR = Mean (RMS_SC)/[Mean (RMS_UT) + Mean (RMS_SCM)]	SC: Semispinalis capitis (deep stabilizer); UT: upper trapezius; SCM: Sternocleidomastoid (superficial movers)
ASI (%)	Coordination	ASI = |RMS_L − RMS_R|/(RMS_L + RMS_R) × 100%	L, R: Bilateral homologous muscle pairs; averaged across three muscle groups
SampEn	Control	SampEn (m, r, N) = −ln [A/B]	m = 2 (template length); r = 0.2 × SD (tolerance); N = signal length; A, B: Template match counts at lengths m and m+1
CoV_trend (%)	Control	CoV_trend = β_1_ from CoV(t) = β_0_ + β_1_t	CoV(t): Coefficient of variation of RMS envelope computed in sliding windows; β_1_: Linear regression slope over time
IFR (%/s)	Fatigue	IFR = (MDF_t2 − MDF_t1)/MDF_t1	t1: First 2.5 s of the 5-s stable segment; t2: second 2.5 s; MDF: Median frequency
MDF_Slope (Hz/s)	Fatigue	MDF_Slope = β_1_ from MDF(t) = β_0_ + β_1_t	MDF(t): Median frequency computed in sequential FFT windows across the 5-s segment; β_1_: Linear regression slope

EMG, electromyography; RMS, root mean square; FFT, fast fourier transform; SD, standard deviation; CCI, formula adapted from Falconer and winter (1985). SampEn calculated following Pincus (1991). All features were computed for each movement direction and averaged per participant for subsequent analyses.

### 2.5 Machine learning analysis

Model evaluation employed Repeated K-Fold cross-validation (n_splits = 5, n_repeats = 10, random_state = 42), yielding 50 independent train-validation partitions across 69 patients. The feature-to-sample ratio (7:69, approximately 1:10) meets the events-per-variable (EPV ≥ 10) criterion recommended for regression modeling (Vabalas et al., 2019). For K-means clustering, the ratio of observations to variables (69:7 ≈ 10:1) satisfies the minimum empirical guideline of ≥ 10 observations per clustering variable, although the sample size represents the lower bound and limits the detection of finer-grained subgroups. Performance metrics included *R* ^2^ (mean ± SD), MAE, and RMSE. To address the modest data-to-model ratio and pre-empt overfitting we combined multiple safeguards: (i) nested GridSearchCV for hyperparameter optimization within each outer training fold, preventing test-set leakage; (ii) shallow trees (max_depth = 4) with subsampling (subsample = 0.8, colsample_bytree = 0.8) and L1/L2 regularization (reg_alpha = 0.1, reg_lambda = 1.0); (iii) permutation testing (n = 1,000) to validate statistical significance of the cross-validated *R* ^2^; (iv) learning-curve analysis (Supplementary Figure S3); and (v) an additional Leave-One-Patient-Out cross-validation (LOPOCV) as an internal-robustness check supplementary to the Repeated K-Fold scheme (Supplementary Table S12). Furthermore, an automated feature-selection sensitivity analysis was performed (LASSO and Recursive Feature Elimination over an expanded 12-feature candidate pool comprising the seven theoretical features plus five basic channel-mean physiological features) to verify that the theory-driven feature set is not predictively inferior to data-driven selections (Supplementary Figure S8; Supplementary Table S13).

Six regression models were compared: multiple linear regression (MLR), Ridge, Lasso, ElasticNet, Random Forest, and XGBoost. All seven EMG features served as inputs with NDI and VAS scores as targets. For all regularized and ensemble models, hyperparameters were optimized using nested GridSearchCV (3-fold CV within each training fold) to prevent data leakage. A permutation test (n = 1,000) validated model significance, and learning curve analysis assessed sample size effects (Supplementary Figure S3). SHAP (SHapley Additive exPlanations) was used to quantify each feature’s contribution to predictions (Supplementary Table S4).

### 2.6 Statistical analysis

Normality was assessed by Shapiro-Wilk test. Continuous variables were expressed as mean ± SD or median (IQR). Between-group comparisons used Mann-Whitney U tests (healthy vs. patients) and Kruskal–Wallis H tests with Dunn’s *post hoc* comparisons (severity groups; Supplementary Tables S6–S7). Effect sizes were calculated using Cohen’s d for two-group comparisons and eta-squared (η^2^) for severity group comparisons. Potential demographic confounding was evaluated through partial Spearman correlations controlling for age, sex, and BMI, and sensitivity analysis comparing XGBoost models with and without demographic covariates (Supplementary Figure S5; Supplementary Table S8).

K-means clustering was employed for unsupervised patient stratification using z-score normalized EMG features. Optimal cluster number was determined by four internal validation indices (Silhouette Score, Calinski-Harabasz Index, Davies-Bouldin Index, Gap Statistic) computed for K = 2–6. Bootstrap stability (n = 1,000 resamples, ARI) and cross-method consistency (hierarchical clustering, DBSCAN) were assessed (Supplementary Figure S6; Supplementary Table S9). Chi-square test evaluated association between cluster membership and clinical severity. All analyses were performed in Python 3.8 (scikit-learn 1.0.2, SHAP 0.41.0, SciPy 1.7.0). Significance was set at α = 0.05 (two-tailed).

## 3 Results

### 3.1 Patient demographic characteristics

Table 2 presents baseline demographic characteristics. Age (43.7 ± 8.7 years), BMI (24.9 ± 3.4 kg/m^2^), disease duration (median 20 months), sex distribution (56.5% male), and VAS scores were comparable across severity groups (all p > 0.39). Only NDI scores differed significantly (p < 0.001), consistent with the NDI-based classification design.

**TABLE 2:** Baseline patient demographic characteristics by severity group.

Variable	Total (n = 69)	Mild (n = 13)	Moderate (n = 23)	Severe (n = 33)	p-value
Age (years)	43.7 ± 8.7	45.7 ± 9.4	42.0 ± 7.9	44.1 ± 9.1	0.398
BMI (kg/m^2^)	24.9 ± 3.4	24.8 ± 3.2	24.5 ± 3.7	25.2 ± 3.3	0.945
Disease duration (months)	20 (11–32)	25 (9–46)	17 (9–24)	20 (13–39)	0.508
VAS score	5.2 ± 1.1	4.9 ± 1.2	5.2 ± 1.0	5.3 ± 1.2	0.453
NDI score	23.2 ± 8.1	11.2 ± 1.9	19.8 ± 2.2	30.4 ± 3.7	<0.001
Sex, male n (%)	39 (56.5%)	9 (69.2%)	13 (56.5%)	17 (51.5%)	0.551

BMI, body mass index; VAS, visual analog scale; NDI, Neck Disability Index. Disease Duration expressed as Median (IQR). Age, BMI, and VAS compared by Kruskal–Wallis test; Sex by Chi-square test. All p-values > 0.05 except NDI (by design).

### 3.2 Quality control results and signal characteristics

**FIGURE 2:**
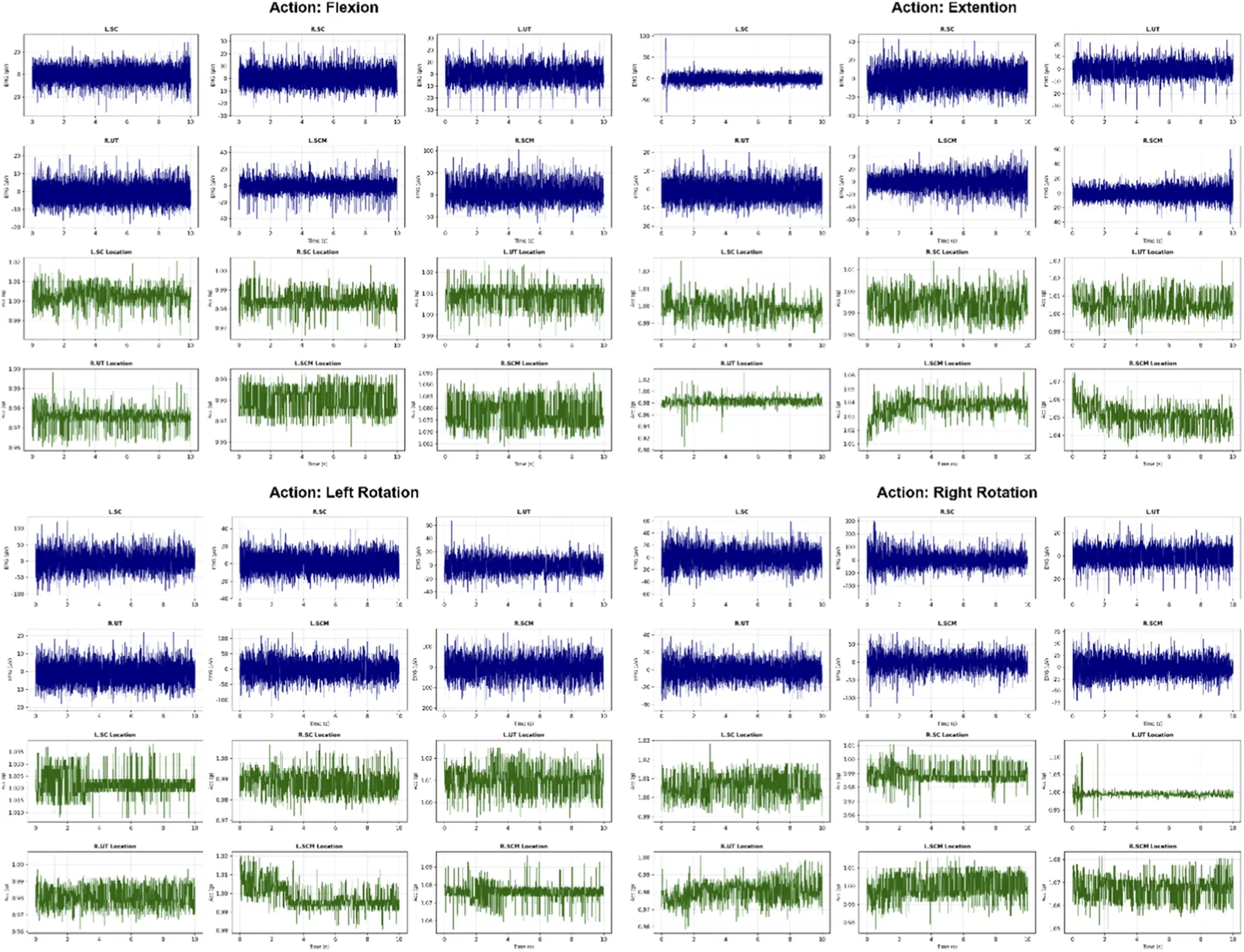
Representative Raw EMG Signals and RMS Envelopes Across Four Movement Directions. Surface EMG recordings from six bilateral cervical muscles during four standardized movements. Upper two rows show raw EMG amplitude (μV) for each channel: L.SC (Left Semispinalis Capitis), R.SC (Right Semispinalis Capitis), L.UT (Left Upper Trapezius), R.UT (Right Upper Trapezius), L.SCM (Left Sternocleidomastoid), R.SCM (Right Sternocleidomastoid). Lower two rows display corresponding normalized RMS envelopes (unitless) representing muscle activation patterns over a 10-s recording period for flexion, extension, left rotation, and right rotation movements.

From 69 participants, 276 segments were extracted across four movement directions (Figure 2). The seven-dimensional quality control system retained 263 segments (95.3% pass rate; composite score mean: 7.12 ± 0.85). Mean SNR was 28.90 ± 5.19 dB with 100% exceeding the 15 dB threshold. Detailed channel-level metrics are provided in Supplementary Figure S1 and Supplementary Table S1. Split-half validation confirmed threshold stability, with a mean pass rate difference between halves of 2.5% ± 1.9%. Bootstrap resampling yielded a 95% CI for the overall pass rate of [92.4%, 97.5%], confirming robustness of the ≥6.0 threshold.

#### 3.2.1 Acquisition architecture validation

Retrospective evaluation of the 276 raw recordings confirmed that the acquisition chain provided stable, integrity-preserving signals throughout the cohort. The mean per-channel internal-noise SNR was 28.42 dB (minimum channel SNR 18.87 dB), with 100% of files exceeding the 20 dB high-quality threshold. No saturation or missing samples were detected (max saturation rate 0.00%; missing-sample rate 0.00%), and the mean absolute baseline-drift slope was 0.046 μV s^-1^. The 50 Hz notch filter delivered a mean attenuation of 7.80 dB and the 100 Hz notch 14.25 dB. Mean maximum inter-channel cross-talk was 0.397, and the augmented Dickey–Fuller stationarity test passed at a 100% rate across all channels and recordings. Channel-level reliability proxied by ICC(3,1) of channel RMS across the four functional tasks ranged from 0.50 to 0.71, consistent with task-dependent activation differences rather than electrode inconsistency. AI-performance sensitivity to controlled perturbations showed only modest degradation: with additive Gaussian noise corresponding to feature SNRs of −3, −6 and −9 dB, NDI XGBoost CV *R* ^2^ changed by < 0.08 from baseline; injection of a simulated ±5% 50 Hz envelope residual produced an analogous small effect. Detailed metrics are reported in Supplementary Figure S7 and Supplementary Table S15.

### 3.3 Distribution of EMG features across movement directions

**FIGURE 3:**
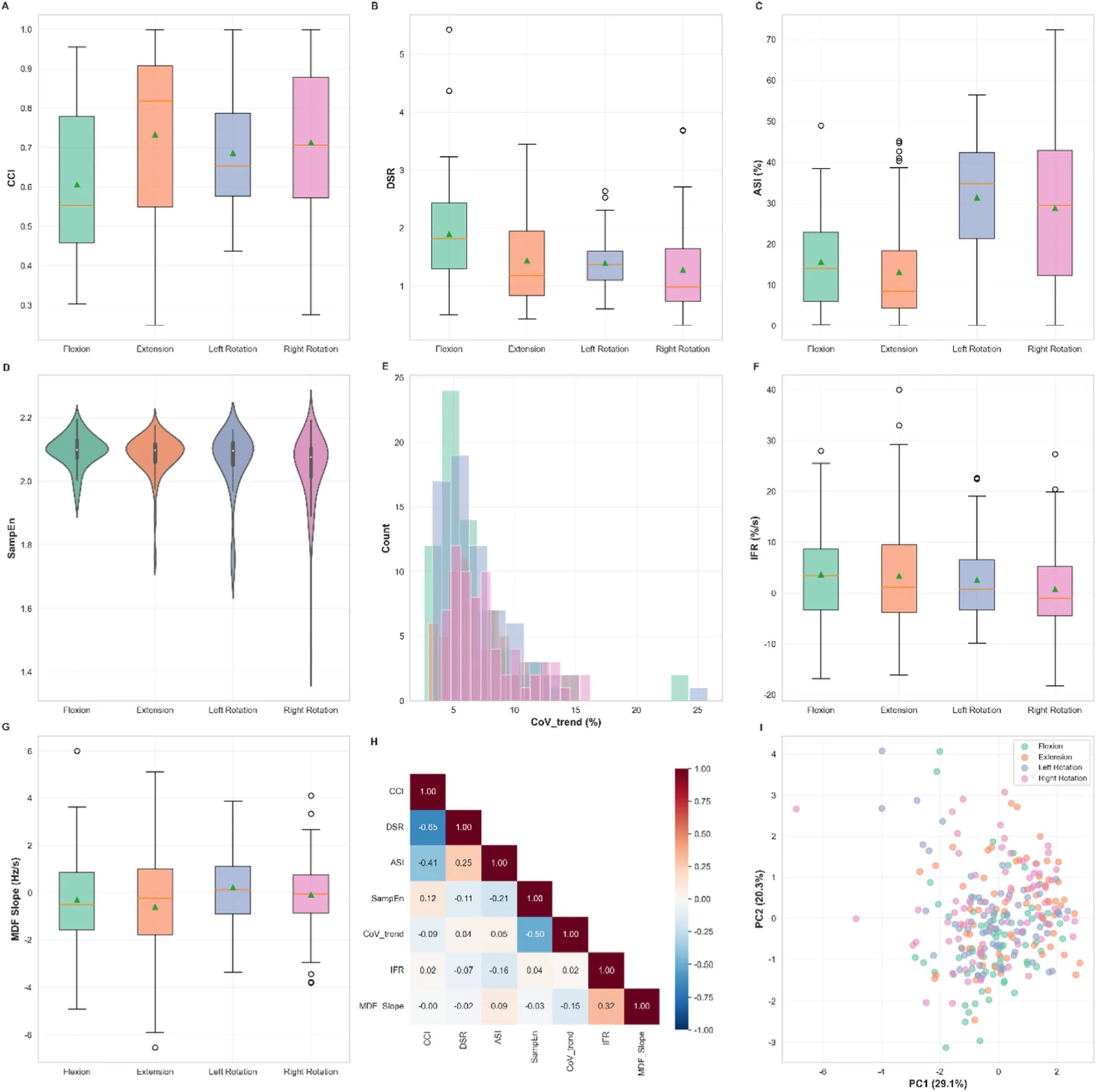
Seven Core EMG Features Extracted from Functional Postural Control Tasks. Biomechanical coordination metrics derived from cervical muscle EMG analysis. **(A)** Co-contraction Index (CCI) by action. **(B)** Deep-to-Superficial Ratio (DSR) by action. **(C)** Asymmetry Index (ASI, %) by action. **(D)** Sample Entropy (SampEn) violin plots by action. **(E)** Coefficient of Variation Trend (CoV_trend, %) histogram across all actions. **(F)** Initial Fatigue Rate (IFR, %/s) by action. **(G)** Median Frequency Slope (MDF_Slope, Hz/s) by action. **(H)** Pearson correlation matrix among the seven features. **(I)** PCA scatter plot showing individual data points colored by movement direction across PC1 (29.1% variance) and PC2 (20.3% variance).

Table 3 and Figure 3 present descriptive statistics and distributions for the seven core features across four movement directions. CCI varied significantly by direction, with extension showing the highest values (0.732 ± 0.217) and flexion the lowest (0.606 ± 0.182, p < 0.001). DSR demonstrated high inter-individual variability (range: 0.65–4.35). ASI was markedly higher during rotational movements (28.8%–31.4%) compared to sagittal movements (13.1%–15.6%). SampEn was consistent across directions (2.044–2.095), while CoV_trend showed a right-skewed distribution (median: 6.7%). PCA explained 49.4% of total feature variance in two components (Figure 3I). Basic physiological characteristics are detailed in Supplementary Table S2; inter-feature correlations are provided in Supplementary Table S3.

**TABLE 3:** Descriptive statistics of seven core EMG features across four cervical movements.

Feature	Flexion	Extension	Left rotation	Right rotation
CCI	0.606 ± 0.182	0.732 ± 0.217	0.686 ± 0.146	0.712 ± 0.190
DSR	1.896 ± 0.885	1.438 ± 0.736	1.394 ± 0.460	1.280 ± 0.733
ASI (%)	15.615 ± 11.098	13.142 ± 12.684	31.387 ± 14.551	28.765 ± 18.984
SampEn	2.095 ± 0.051	2.084 ± 0.062	2.065 ± 0.100	2.044 ± 0.110
CoV_trend (%)	6.518 ± 3.843	7.244 ± 2.949	7.211 ± 3.519	7.787 ± 2.961
IFR (%/s)	3.629 ± 10.189	3.373 ± 11.754	2.542 ± 7.718	0.757 ± 8.311
MDF_Slope (Hz/s)	−0.307 ± 2.016	−0.608 ± 2.642	0.215 ± 1.602	−0.093 ± 1.585

CCI, Co-contraction Index; DSR, Deep-to-Superficial Ratio; ASI, asymmetry index; SampEn = Sample Entropy; CoV_trend = Coefficient of Variation trend of RMS, envelope; IFR, initial fatigue rate; MDF_Slope = Median Frequency Slope. Data presented as mean ± SD, for n = 65–69 patients per movement.

### 3.4 Machine learning model performance and feature importance

Table 4 presents comparative model performance under identical cross-validation conditions. XGBoost achieved the highest predictive performance for NDI (CV *R* ^2^ = 0.449 ± 0.182, MAE = 4.658 ± 0.737), outperforming all linear models by 13%–16%. Random Forest ranked second (CV *R* ^2^ = 0.417 ± 0.190). A permutation test confirmed statistical significance (p = 0.001). Learning curve analysis indicated that performance may benefit from larger samples (Supplementary Figure S3). All models showed poor VAS prediction (CV *R* ^2^ < 0); subsequent analysis focused on NDI. Specifically, all four linear models yielded negative CV *R* ^2^ values (range: −0.168 to −0.110), indicating predictions worse than the sample mean. XGBoost and Random Forest achieved near-zero CV *R* ^2^ (0.030 ± 0.652 and 0.089 ± 0.548, respectively), with MAE of approximately 0.80–0.92 relative to a VAS SD of 1.1, indicating that prediction errors approached the scale of natural VAS variability. Full VAS model performance metrics are reported in Supplementary Table S10.

**TABLE 4:** Comparative model performance for NDI prediction.

Model	CV *R* ^2^ (Mean ± SD)	CV MAE (Mean ± SD)	CV RMSE (Mean ± SD)
MLR	0.392 ± 0.219	4.820 ± 0.760	5.856 ± 0.918
Ridge	0.387 ± 0.210	4.884 ± 0.750	5.891 ± 0.872
Lasso	0.389 ± 0.212	4.847 ± 0.769	5.883 ± 0.919
ElasticNet	0.390 ± 0.207	4.866 ± 0.757	5.885 ± 0.894
Random forest	0.417 ± 0.190	4.904 ± 0.792	5.774 ± 0.746
**XGBoost**	**0.449 ± 0.182**	**4.658 ± 0.737**	**5.607 ± 0.766**

CV, cross-validation; *R* ^2^ = coefficient of determination; MAE, mean absolute error; RMSE, root mean square error; SD, Standard Deviation; MLR, Multiple Linear Regression. All models evaluated on n = 69 patients using RepeatedKFold (n_splits = 5, n_repeats = 10, random_state = 42). Bold indicates best performance.

**FIGURE 4:**
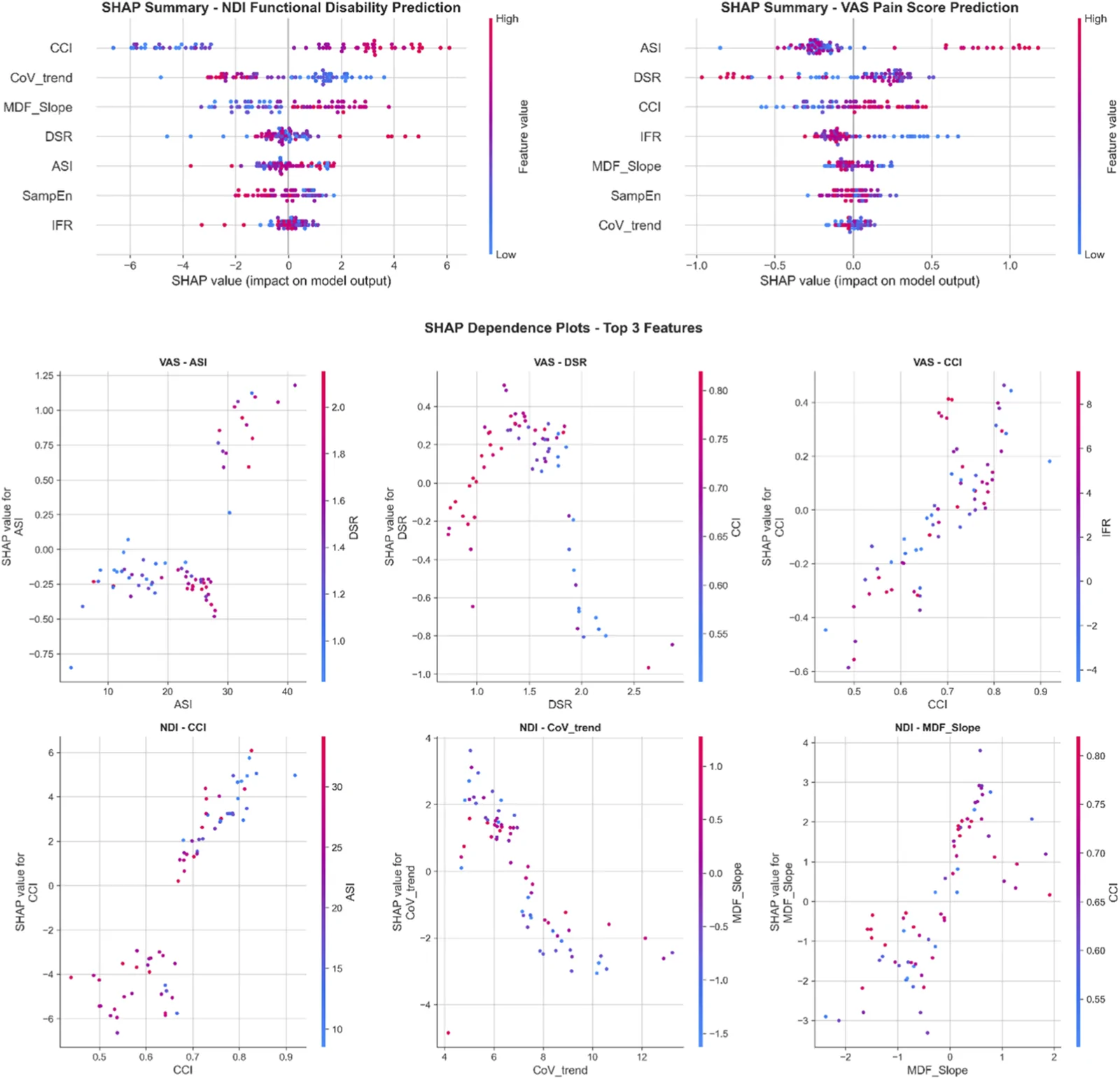
SHAP Analysis for Clinical Outcome Prediction. Feature importance quantified using SHAP values. Upper panels: Summary plots ranking features by mean absolute SHAP value for NDI (left) and VAS (right) predictions. Color indicates feature magnitude. Lower panels: Dependence plots for top three features showing relationships between feature values, SHAP values, and interaction effects, with secondary features color-coded.

SHAP analysis identified CCI as the most influential NDI predictor (mean |SHAP|: 3.68), followed by CoV_trend (1.74) and MDF_Slope (1.56). Dependence plots revealed a nonlinear CCI-NDI relationship with an inflection point at CCI ≈ 0.67 (95% Bootstrap CI: 0.50–0.76; piecewise regression *R* ^2^ = 0.443 vs. linear *R* ^2^ = 0.319, F = 7.24, p = 0.002), beyond which disability increased sharply (Figure 4). Feature interaction effects were modest (strongest: CCI × IFR, r = 0.32). Confounding analysis confirmed that EMG-NDI associations were independent of demographics: partial correlations controlling for age, sex, and BMI showed maximum change <0.1%, and adding demographic covariates to XGBoost decreased performance (CV *R* ^2^: 0.449 → 0.380; Supplementary Figure S5; Supplementary Table S8). Internal robustness was further evaluated by Leave-One-Patient-Out cross-validation (LOPOCV), which yielded NDI *R* ^2^ = 0.524 (95% bootstrap CI: 0.328–0.658; RMSE = 5.54; MAE = 4.62; Pearson r = 0.725), corroborating the Repeated K-Fold estimate. SHAP feature-importance rankings were highly stable across the 69 LOPO folds (Kendall’s W = 0.960 for NDI), indicating that the identified predictors are not artefacts of fold-specific data partitions (Supplementary Table S12). The automated feature-selection sensitivity analysis confirmed that the theory-driven 7-feature set (CV *R* ^2^ = 0.475 ± 0.199; 95% CI 0.418–0.530) performed comparably to LASSO-selected (0.469 ± 0.203; 95% CI 0.410–0.525) and RFE-top-7 (0.440 ± 0.198; 95% CI 0.385–0.494) subsets drawn from a broader 12-feature pool, with all 7 theoretical features retained by LASSO and 6/7 retained by RFE (Supplementary Figure S8; Supplementary Table S13).

### 3.5 EMG features across disease severity levels

**FIGURE 5:**
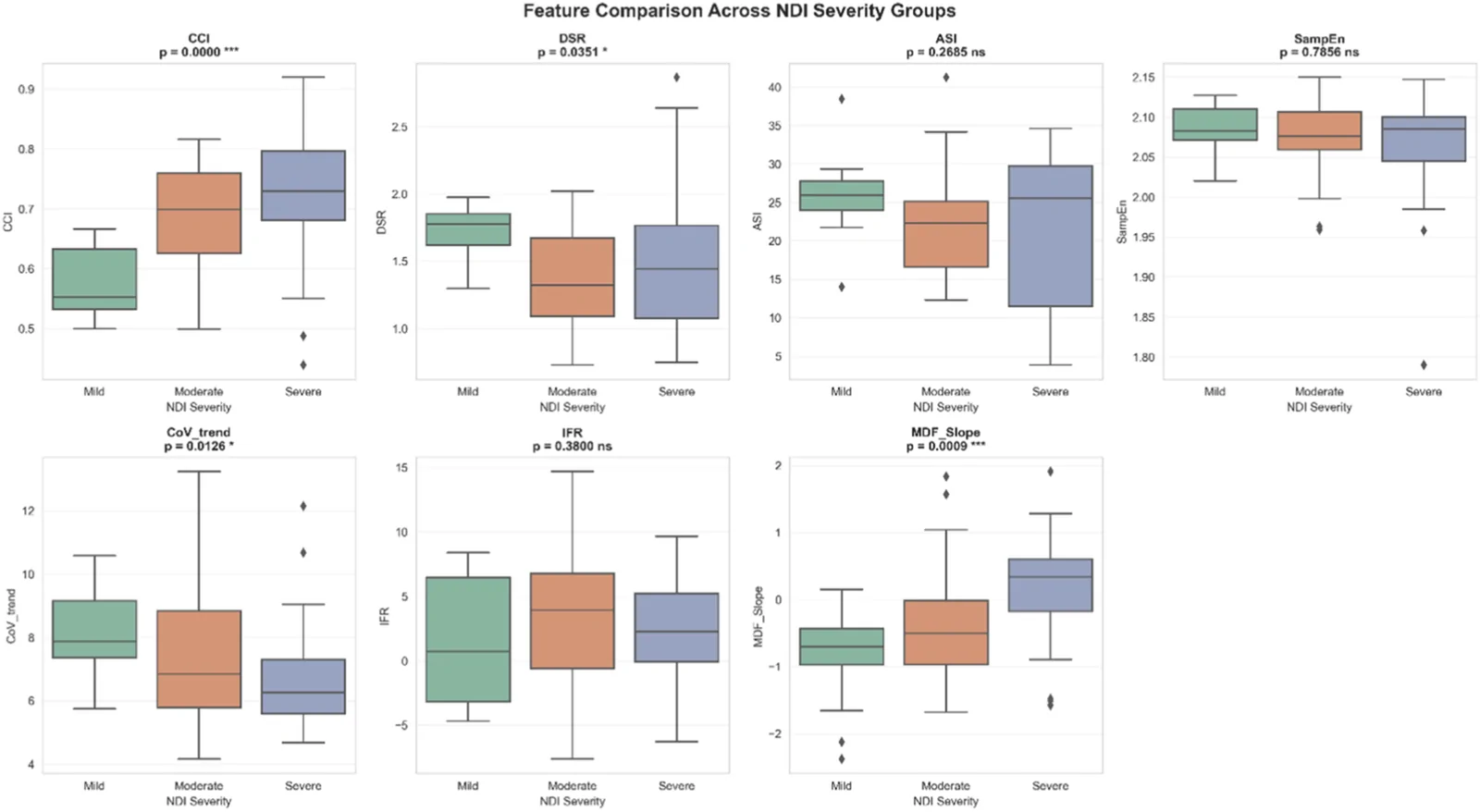
EMG Feature Comparison Across NDI Severity Groups. Box plots comparing seven features across three NDI severity categories: Mild (NDI: 0–14), Moderate (NDI: 15–24), and Severe (NDI ≥25). Features shown: CCI (p < 0.0001), DSR (p = 0.0351), ASI (p = 0.2685, ns), SampEn (p = 0.7856, ns), CoV_trend (p = 0.0126), IFR (p = 0.3800, ns), MDF_Slope (p = 0.0009). Statistical significance determined by Kruskal–Wallis test (p < 0.05, **p < 0.001, ns = not significant).

Four features showed significant between-group differences (Table 5; Figure 5). CCI progressively increased from mild to severe groups (0.57 ± 0.06 vs. 0.73 ± 0.10; p < 0.001, η^2^ = 0.289). DSR was significantly higher in mild than moderate patients (1.73 vs. 1.37; p = 0.033, η^2^ = 0.071). MDF_Slope demonstrated progressive changes: mild (−0.86 Hz/s), moderate (−0.34 Hz/s), severe (0.16 Hz/s; severe vs. mild p < 0.001, η^2^ = 0.182). CoV_trend decreased with severity (p = 0.013, η^2^ = 0.102). ASI, SampEn, and IFR showed no significant differences (all p > 0.25). Post-hoc results are detailed in Supplementary Table S6; 95% confidence intervals for key features across severity groups are provided in Supplementary Table S11.

**TABLE 5:** Comparison of EMG features across NDI severity groups.

Feature	Mild (n = 13)	Moderate (n = 23)	Severe (n = 33)	H	p-value	η^2^
CCI	0.573 ± 0.058	0.688 ± 0.084	0.726 ± 0.100	21.078	<0.001	0.289
DSR	1.725 ± 0.193	1.372 ± 0.401	1.505 ± 0.513	6.697	0.035	0.071
ASI (%)	25.778 ± 5.413	21.701 ± 7.211	21.196 ± 9.746	2.63	0.269	0.010
SampEn	2.084 ± 0.029	2.073 ± 0.050	2.067 ± 0.067	0.483	0.786	0.000
CoV_trend (%)	8.160 ± 1.388	7.468 ± 2.473	6.614 ± 1.629	8.755	0.013	0.102
IFR (%/s)	1.244 ± 4.723	3.609 ± 5.389	2.379 ± 4.116	1.935	0.380	0.000
MDF_Slope (Hz/s)	−0.859 ± 0.777	−0.340 ± 0.937	0.161 ± 0.797	14.016	<0.001	0.182

NDI, Neck Disability Index. Severity groups: Mild (0–14, n = 13), Moderate (15–24, n = 23), Severe (≥25, n = 33). H = Kruskal–Wallis H statistic; η^2^ = eta-squared effect size. CCI, Co-contraction Index; DSR, Deep-to-Superficial Ratio; ASI, asymmetry index; SampEn = Sample Entropy; CoV_trend = Coefficient of Variation trend of RMS, envelope; IFR, initial fatigue rate; MDF_Slope = Median Frequency Slope. Data presented as mean ± SD.

### 3.6 Identification of neuromuscular compensatory states

K-means clustering identified K = 2 as optimal, supported by convergent Silhouette Score (0.199) and Calinski-Harabasz Index (18.97) maxima (Supplementary Figure S6; Supplementary Table S9). Bootstrap stability analysis yielded a mean ARI of 0.585 ± 0.325, indicating moderate reproducibility.

**FIGURE 6:**
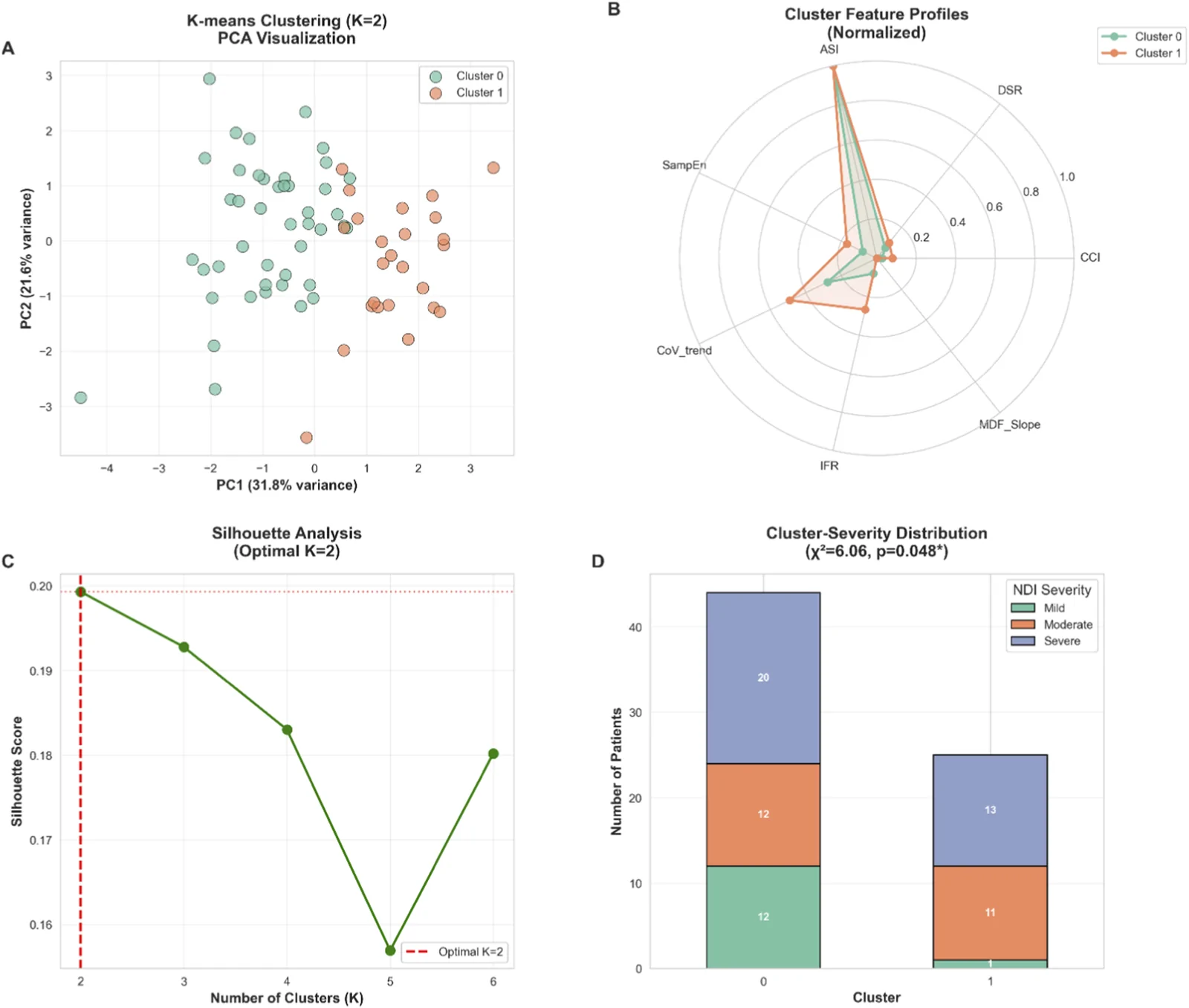
Unsupervised Clustering Revealing Neuromuscular Compensatory States. K-means clustering (K = 2) identifying patient subgroups. **(A)** PCA visualization showing cluster separation across PC1 (31.8% variance) and PC2 (21.6% variance). **(B)** Normalized radar plot comparing mean feature profiles between Cluster 0 (“Asymmetric Compensation”, teal) and Cluster 1 (“Global Stiffening”, coral). **(C)** Silhouette analysis validating optimal K = 2 (highest score = 0.20). **(D)** Stacked bar chart showing cluster distribution across NDI severity categories (χ^2^ = 6.06, p = 0.048).

Two candidate neuromuscular states were identified (Table 6; Figure 6). Cluster 0 (n = 44, 63.8%), termed “Asymmetric Compensation,” was characterized by elevated ASI (26.7%), preserved DSR (1.744), low CCI (0.635), and near-flat MDF_Slope (−0.079 Hz/s), indicating lateralized load redistribution with maintained deep stabilizer function and low metabolic cost. Cluster 1 (n = 25, 36.2%), termed “Global Stiffening,” exhibited high CCI (0.771), reduced DSR (1.077), low ASI (14.4%), and steep MDF_Slope (−0.408 Hz/s), indicating global co-contraction with deep stabilizer inhibition and accelerated fatigue. SampEn and CoV_trend were comparable between clusters. PCA separated the two states along PC1 (31.8% variance; Figure 6A). Cluster membership was significantly associated with NDI severity (χ^2^ = 6.06, p = 0.048), with Cluster 1 containing a higher proportion of severe patients (52% vs. 27%; Figure 6D).

**TABLE 6:** Characteristics of patient subtypes identified by K-means clustering.

Characteristic	Cluster 0 (n = 44)	Cluster 1 (n = 25)	Interpretation
VAS score	5.239 ± 1.244	5.068 ± 0.931	Comparable pain intensity
NDI score	21.932 ± 8.445	25.520 ± 7.024	↑Greater functional disability in cluster 1
CCI	0.635 ± 0.091	0.771 ± 0.058	↑Global co-contraction in cluster 1
DSR	1.744 ± 0.340	1.077 ± 0.239	↑Superficial compensation in cluster 1
ASI (%)	26.703 ± 6.115	14.351 ± 5.400	↑Spatial asymmetry in cluster 0
SampEn	2.065 ± 0.061	2.083 ± 0.044	Preserved motor complexity
CoV_trend (%)	7.385 ± 2.023	6.846 ± 1.902	Comparable temporal stability
IFR (%/s)	2.033 ± 4.658	3.530 ± 4.696	↑Early rapid fatigue in cluster 1
MDF_Slope (Hz/s)	−0.079 ± 0.886	−0.408 ± 0.954	↑Sustained fatigue progression in cluster 1

VAS, Visual Analog Scale (0–10); NDI, Neck Disability Index (0–50). K-means clustering performed on standardized EMG, features. Arrows (↑) indicate features with between-cluster differences >0.5 SD., Cluster 0 = “Asymmetric Compensation”; Cluster 1 = “Global Stiffening”. CCI, Co-contraction Index; DSR, Deep-to-Superficial Ratio; ASI, asymmetry index; SampEn = Sample Entropy; CoV = Coefficient of Variation of RMS, trend; IFR, initial fatigue rate; MDF_Slope = Median Frequency Slope; RMS, Root Mean Square. Data presented as mean ± SD.

## 4 Discussion

This study established a seven-feature EMG framework across three functional dimensions, quantified feature predictive values for neck pain clinical outcomes, and identified two exploratory neuromuscular compensatory states. Three principal findings emerged: (1) CCI, CoV_trend, and MDF_Slope were the primary predictors of functional disability (NDI); (2) disease severity was associated with systematic EMG feature changes; and (3) patients could be stratified into two candidate compensatory states — “Asymmetric Compensation” and “Global Stiffening” — with distinct clinical profiles.

### 4.1 Selection rationale and clinical significance of seven core features

The seven features represent established indicators in EMG motor control and fatigue research (Hodges and Smeets, 2015; Madeleine et al., 2008; Enoka and Duchateau, 2016), capturing higher-order functional information beyond basic amplitude or spectral parameters (Sanderson et al., 2019; Farina et al., 2008). The coordination dimension (CCI, DSR, ASI) quantifies antagonist co-activation, deep-to-superficial balance, and bilateral symmetry (Hug, 2011); the control dimension (SampEn, CoV_trend) reflects motor complexity and postural stability (Pincus, 1991); and the fatigue dimension (IFR, MDF_Slope) captures both early and sustained fatigue through frequency-domain indices (Merletti et al., 2001). Evaluating functional endurance during submaximal tasks aligns more closely with daily activity demands than MVIC protocols.

### 4.2 Predictive value of EMG features for clinical outcomes

XGBoost consistently outperformed all linear models under identical evaluation conditions, supporting nonlinear EMG-NDI relationships. Ensemble tree methods capture interaction effects and threshold phenomena that linear frameworks cannot model (Lundberg and Lee, 2017; Chen and Guestrin, 2016). The comparable performance of all four linear models suggests that the performance gap reflects a fundamental limitation of linearity rather than differences between regularization strategies, consistent with Hodges and Smeets’ (Hodges and Smeets, 2015) characterization of pain-motor interactions as complex feedback loops.

SHAP analysis provided clinically interpretable insights. The CCI inflection at approximately 0.67 (95% CI: 0.50–0.76) identifies a threshold beyond which disability escalates sharply, relevant to clinical warning indicators. However, the wide CI reflects substantial uncertainty, and this threshold requires prospective validation. CoV_trend emerged as a novel disability surrogate, likely reflecting compromised sensorimotor control (van den Hoorn et al., 2015). The negative MDF_Slope-NDI association is consistent with known fatigue-disability relationships (Barbero et al., 2012). For pain prediction, ASI’s contribution aligns with evidence linking asymmetric activation to altered spinal loading (Caneiro et al., 2010).

Feature discriminative capacity was confirmed by healthy volunteer comparisons (five of seven features with large effect sizes) and confounding analysis showing virtually unchanged EMG-NDI correlations after controlling for demographics (maximum change <0.1%; Supplementary Table S8), consistent with Panjabi’s spinal stability framework (Panjabi, 1992).

### 4.3 Limitations of EMG-Based pain intensity prediction

All models failed to predict VAS scores in the Repeated K-Fold scheme (CV *R* ^2^ ≤ 0 for all linear models; ≈ 0 for ensemble models; Supplementary Table S10), and LOPOCV yielded only modest predictive signal (*R* ^2^ = 0.286, 95% CI: −0.005–0.475). Three complementary reasons account for this outcome. First (mechanism), pain intensity and functional disability are driven by partially distinct pathways: surface EMG captures motor output and muscle-function dimensions, whereas VAS reflects a subjective sensory–affective experience modulated by psychological, emotional and cognitive factors that lie outside the EMG signal space. Second (statistical), an *a priori* power analysis (n = 69, α = 0.05, power = 0.80, observed VAS SD ≈ 1.14) showed that the minimum detectable per-feature |r| under our sample size is 0.332 and the minimum detectable multivariable *R* ^2^ (7 predictors) is 0.189. All observed feature–VAS correlations fell below the 0.332 threshold, and the measurement-noise ceiling derived from a conservative VAS test–retest ICC of 0.85 imposes a hard upper bound (*R* ^2^_max = 0.85) on any feature set; together these parameters demonstrate that the present cohort is underpowered to detect EMG–VAS effects of the magnitude reported in the literature (Supplementary Table S14). Third (literature), prior EMG studies in chronic neck and shoulder pain consistently report weak EMG–pain intensity associations (|r| ≈ 0.14–0.25 across six representative studies summarized in Supplementary Table S14), corroborating that the EMG signal space is, in general, a stronger correlate of functional disability than of subjective pain intensity. Together these considerations support reframing sEMG-based clinical analytics toward functional disability prediction rather than direct pain quantification.

### 4.4 EMG feature evolution patterns across severity levels

CCI progressively increased across severity levels, consistent with the defensive stiffness hypothesis (Falla et al., 2013). MDF_Slope showed a nonlinear pattern—declining in mild-moderate patients but increasing in severe patients—possibly reflecting altered motor unit recruitment or fiber type adaptations (Falla and Farina, 2008; Farina et al., 2004). DSR was highest in mild patients and declined significantly in moderate patients, suggesting early preservation of deep stabilizer function that deteriorates with progression. These patterns suggest that different severity stages may benefit from differentiated rehabilitation approaches.

### 4.5 Two exploratory neuromuscular compensatory states

Unsupervised clustering suggested two candidate neuromuscular states that align with compensatory strategies described in the pain-motor adaptation literature.

The Asymmetric Compensation state (Cluster 0, 63.8%) exhibited elevated ASI with preserved DSR and low CCI, suggesting spatial load redistribution through lateralized recruitment while maintaining deep stabilizer engagement (Hodges and Smeets, 2015; O’Sullivan, 2005). The near-flat MDF_Slope indicates low metabolic cost, as deep stabilizers continue functioning and superficial muscles are not forced into sustained postural roles. The balanced NDI distribution (mild 27%, moderate 27%, severe 46%) is consistent with a relatively adaptive strategy.

The Global Stiffening state (Cluster 1, 36.2%) showed high CCI with reduced DSR and low ASI, consistent with Panjabi’s model (Panjabi, 1992): deep stabilizer inhibition (Schomacher and Falla, 2013; Falla et al., 2004b) forces superficial muscles into sustained co-contraction, producing bilateral bracing. The steeply negative MDF_Slope and elevated IFR indicate that these superficial muscles undergo rapid fatigue when performing sustained tonic activity contrary to their fiber-type characteristics (Falla and Farina, 2008), suggesting a mechanistic pathway: deep muscle inhibition → superficial compensation → co-contraction → accelerated fatigue → functional disability (Schomacher and Falla, 2013; Falla et al., 2004b; Falla and Farina, 2008). The higher proportion of severe patients (52% vs. 46%) is consistent with this being a more maladaptive strategy.

The two states represent divergent solutions to cervical instability: spatial load redistribution with preserved deep stabilizers versus rigid global bracing with deep stabilizer inhibition. These divergent profiles generate testable hypotheses for future controlled trials—for example, whether Asymmetric Compensation patients benefit preferentially from symmetry-oriented training and Global Stiffening patients from deep stabilizer reactivation and graded motor exposure—none of which were evaluated in the present cross-sectional study.

Bootstrap stability analysis yielded a mean ARI of 0.585 ± 0.325 (95% CI including negative values), and cross-method validation (K-means vs. hierarchical ARI = 0.247) indicated only moderate reproducibility. These results, together with the moderate silhouette score (0.199), suggest that the two-state solution should be regarded as exploratory. Neuromuscular compensation likely exists along a continuum (Dankaerts et al., 2006; Luomajoki et al., 2010), and larger samples are needed to determine whether these represent discrete states or regions along a spectrum. Several considerations support the use of K-means despite the modest Silhouette Score of 0.199 and bootstrap ARI confidence interval crossing zero. First, centroid-based clustering provides interpretable phenotype prototypes that map directly to clinically actionable feature profiles, an essential property for translational use. Second, K-means is well matched to the continuous, standardized feature space here (z-scored EMG indices) and is robust under our n = 69 sample size relative to distribution-sensitive alternatives. Third, cross-method validation explicitly tested whether stronger structure could be recovered by different algorithms: hierarchical agglomerative clustering yielded only weak agreement (ARI = 0.247) and DBSCAN identified a single cluster (Supplementary Figure S6), indicating that the limited cluster separability reflects intrinsic properties of the data rather than a suboptimal algorithm choice. Accordingly, the two-state solution is reported as a hypothesis-generating phenotypic delineation, not a diagnostic classifier; prospective, multi-centre cohorts are required to corroborate, refine or refute this provisional partition.

### 4.6 Position relative to hybrid physical–computational approaches

The present framework adopts a theory-informed, data-driven design: seven mechanistically grounded EMG features (CCI, DSR, ASI, SampEn, CoV_trend, IFR, MDF_Slope) encode coordination, motor control and fatigue, and are mapped onto NDI by an interpretable gradient-boosted ensemble with SHAP attributions. Recent work in biomedical electronic-device monitoring illustrates that combining structured analytical methods with machine-learning components can improve interpretability and robustness (Vincenzi et al., 2026b). In the present clinical context, we did not incorporate explicit finite-element (FEM) musculoskeletal modelling because subject-specific cervical FEM pipelines still require imaging input, individualized tissue-parameter calibration and substantial mesh-generation effort that are not yet realistic at the 69-patient feasibility scale of this study. Coupling the proposed EMG phenotypes with subject-specific cervical FEM models—to derive physics-informed, individualized rehabilitation prescriptions—therefore represents a natural and well-defined extension that we plan to pursue in subsequent work.

### 4.7 Clinical feasibility considerations

The proposed framework has practical strengths: sEMG is non-invasive, low-cost, and increasingly portable; the protocol requires approximately 10 min per patient; and the feature extraction uses established methods implementable in automated software. However, electrode placement and signal quality control remain operator-dependent steps requiring training. The machine learning pipeline requires computational infrastructure not yet standard in clinical settings, though cloud-based sEMG platforms are emerging. Inter-session and inter-operator reproducibility requires formal evaluation, and decision thresholds (e.g., CCI ≈ 0.67) need multi-center prospective validation.

### 4.8 Study limitations

Several limitations warrant consideration. First, this single-center, cross-sectional study (n = 89, 69 patients) has a modest sample size that limits statistical power for subgroup analyses, constrains the stability of clustering solutions, and precludes causal inference regarding EMG feature changes and disease progression. No external validation cohort was available; all predictive thresholds (e.g., CCI inflection at 0.67), clustering boundaries, and feature norms were derived and evaluated within the same dataset and require multi-center prospective validation before clinical adoption. Second, formal intra- and inter-operator reliability for electrode placement and sEMG features was not assessed. Although standardized anatomical landmarks and trained operators were used, reproducibility of the proposed features across operators and sessions remains to be established. Third, the 5-s analysis window, although selected algorithmically for signal stationarity, captures initial fatigue dynamics rather than sustained fatigue progression. Fourth, the clustering solution showed only moderate bootstrap stability (ARI = 0.585 ± 0.325, 95% CI including negative values), and the identified compensatory states should be considered exploratory phenotypes rather than definitive clinical categories. Although LOPOCV was added as a stricter internal-robustness check (Supplementary Table S12), cross-validation alone—whether K-fold, repeated K-fold, or leave-one-out—cannot substitute for external validation in clinical applications. The reported predictive performance, severity thresholds and phenotypic delineations should therefore be regarded as internally consistent but externally unverified. Prospective multi-centre data collection with site-independent operators, scanners and patient mixes, ideally pre-registered, is required before any of the identified features, thresholds or compensatory states are translated into routine clinical use. Future directions include external validation on independent cohorts, longitudinal monitoring of EMG phenotypes across rehabilitation, and integration with subject-specific biomechanical (finite-element) musculoskeletal modelling to enable physics-informed, individualized intervention planning.

## Data Availability

The raw data supporting the conclusions of this article will be made available by the authors, without undue reservation.
