# Supplementary material for: Machine learning analysis of surface electromyography for quantifying neuromuscular compensatory profiles and associated functional disability in neck pain
Source: Front Bioeng Biotechnol. 2026 Aug 11;14:1804580. doi: 10.3389/fbioe.2026.1804580 (PMC13503558; doi:10.3389/fbioe.2026.1804580)
Supplement: Supplementary file 1 [file Supplementaryfile1.docx]

**Figure S1.** Signal Quality Control Based on Internal Noise SNR Methodology. Multi-dimensional EMG quality assessment. A. Internal Noise SNR distribution (450–900 Hz) across six channels with quality thresholds: Excellent (≥20 dB), Good (15–20 dB), Warning (10–15 dB), Fail (<10 dB). B. Crest Factor distribution with acceptable range (2–10). C. RMS Coefficient of Variation distribution for MVIC stability assessment (threshold <30%). D. Channel-wise quality summary showing overall pass rate ≥15 dB: 100.0% across 260 samples.

**Figure S2.** Intelligent Window Segmentation with Seven-Dimensional Quality Control. Automated selection of stable movement epochs. A. Example showing 5-second optimal window (green) determined by composite acceleration and RMS envelope criteria. B. Quality score distribution (mean=7.12, median=7.25, pass rate=95.3%). C. Window start time by action type. D. Statistics summary table. E. Normalized radar plot of seven quality metrics. F. Sample status distribution: 95.3% pass (263 samples), 4.7% warning (13 samples).

**Figure S3.** Multi-Model Comparison for NDI Prediction Using Repeated K-Fold Cross-Validation. (A) Cross-validation R² (Mean ± SD) across six regression models evaluated under identical Repeated K-Fold conditions (5 folds × 10 repeats = 50 evaluations). XGBoost achieved the highest CV R² (0.449 ± 0.182), followed by Random Forest (0.417 ± 0.190), while all four linear models performed comparably (range: 0.387–0.392). (B) Learning curve analysis for XGBoost showing training score (stable at ≈0.99) and cross-validation score (increasing from ≈0.07 to ≈0.41) as a function of training set size. (C) Permutation test distribution (n=1,000) for XGBoost NDI prediction. The observed CV R² (0.436) significantly exceeds the null distribution (p = 0.001).

**Figure S4.** Comparison of EMG Features Between Healthy Volunteers and Cervical Spondylosis Patients. (Top row) Box plots comparing 20 healthy controls and 69 patients for seven features. Six of seven features showed significant differences (Mann-Whitney U test): CCI (p<0.001, d = −2.39), DSR (p<0.001, d = −0.92), ASI (p<0.001, d = −1.78), SampEn (p<0.001, d = 2.05), CoV_trend (p<0.001, d = −1.70), IFR (p=0.017, d = −0.56). MDF_Slope was not significant (p=0.464, d = −0.14). (Bottom row) Box plots across four groups (Healthy, Mild, Moderate, Severe) with Kruskal-Wallis/Dunn’s post-hoc tests. Note: Healthy volunteers (n=20) were matched to patients for age, sex, and BMI (all p > 0.05).

**Figure S5.** Partial Correlation Analysis Controlling for Demographic Confounders. Comparison of zero-order Spearman correlations and partial correlations (controlling for age, sex, BMI) between seven EMG features and NDI scores. Controlling for demographic variables produced 0% change in correlation coefficients for all seven features, confirming EMG-NDI association independence. Significantly correlated features: CCI (ρ = 0.602, p<0.001), CoV_trend (ρ = −0.375, p=0.002), MDF_Slope (ρ = 0.318, p=0.008).

**Figure S6.** Comprehensive Clustering Validation Analyses. (A) Internal validation indices across K=2 to 6: Silhouette Score and Calinski-Harabasz Index both peak at K=2. (B) Bootstrap stability distribution (n=1,000): Mean ARI = 0.585 ± 0.325; 95% CI = [−0.035, 1.000]. (C) t-SNE visualization (perplexity=15) colored by K-means cluster. (D) Cross-method comparison: K-means vs. Hierarchical ARI = 0.247; DBSCAN identified only 1 cluster.

**Figure S7. EMG Acquisition Architecture Validation — Retrospective Robustness Analysis.** Four-panel validation of the EMG acquisition chain across 276 recordings, presented as a 2 × 2 grid. A. Per-channel internal-noise signal-to-noise ratio (SNR; dB) box plot across six bilateral cervical muscle channels (L.SC, R.SC, L.UT, R.UT, L.SCM, R.SCM); horizontal dashed line indicates the 15 dB quality threshold. B. Per-channel 50 Hz notch filter attenuation (dB) demonstrating consistent power-line suppression. C. Distribution of maximum inter-channel cross-talk (|Pearson r|) across recordings (histogram with KDE overlay; mean indicated). D. AI-performance robustness curve: NDI XGBoost cross-validated R² under five controlled perturbations (baseline; additive Gaussian noise at feature SNR −3, −6 and −9 dB; ±5% 50 Hz residual envelope perturbation); error bars indicate cross-validation SD. Note: Two additional integrity checks — ADC saturation rate and augmented Dickey–Fuller stationarity — are not visualised because the cohort-wide values are uniform: saturation rate was 0.000% across all 276 recordings × 6 channels (no sample reached the 95% full-scale ADC threshold), and the ADF stationarity test passed at a 100% rate across all channels and recordings. These uniform "ideal" values are reported in Table S15 and confirm signal integrity at the hardware level.

**Figure S8. Automated vs. Theory-Driven Feature Selection for NDI Prediction.** Comparison across an expanded 12-feature candidate pool (7 theoretical + 5 basic physiological features). A. Feature ranking by LASSO absolute coefficient (sorted descending); theoretical features highlighted; star markers indicate features retained by Recursive Feature Elimination (RFE-top-7). B. Cross-validated R² for NDI prediction under three feature-selection schemes: theory-driven (7 features), LASSO (auto), and RFE-top-7 (XGBoost), with 95% confidence intervals from bootstrap (n=2,000 resamples).

**Table S1. Preprocessing and Quality Control Statistics**

| **Channel** | **Mean SNR** | **SD SNR** | **Excellent %** | **Good %** | **Warning %** | **Fail %** | **Mean CF** | **CF Pass %** | **Mean RMS CoV** | **CoV Pass %** |
| --- | --- | --- | --- | --- | --- | --- | --- | --- | --- | --- |
| L.SC | 28.45 | 4.48 | 100 | 0 | 0 | 0 | 5.67 | 96.9 | 0.239 | 83.8 |
| R.SC | 28.31 | 5.09 | 99.6 | 0.4 | 0 | 0 | 5.65 | 99.2 | 0.220 | 83.1 |
| L.UT | 27.72 | 3.99 | 100 | 0 | 0 | 0 | 6.04 | 98.8 | 0.319 | 53.5 |
| R.UT | 27.63 | 5.93 | 100 | 0 | 0 | 0 | 5.16 | 99.6 | 0.186 | 93.8 |
| L.SCM | 30.63 | 5.49 | 100 | 0 | 0 | 0 | 6.15 | 96.2 | 0.273 | 75.0 |
| R.SCM | 30.66 | 4.97 | 100 | 0 | 0 | 0 | 5.98 | 98.5 | 0.236 | 81.9 |
| Overall | 28.90 | 5.19 | 99.9 | 0.1 | 0 | 0 | 5.78 | 98.2 | 0.246 | 78.5 |

*SNR = Signal-to-Noise Ratio; CF = Crest Factor; RMS CoV = Root Mean Square Coefficient of Variation. Quality thresholds: Excellent (SNR≥20 dB), Good (15≤SNR<20 dB), Warning (10≤SNR<15 dB), Fail (SNR<10 dB).*

**Table S2. Basic Physiological Characteristics of EMG Signals Across Cervical Movements**

*Data presented as mean ± SD for n=65 patients per movement. Values extracted from 5-second stable segments.*

| **Feature-Channel** | **Flexion** | **Extension** | **Left Rotation** | **Right Rotation** |
| --- | --- | --- | --- | --- |
| RMS (μV) |  |  |  |  |
| L.SC | 4.31±1.82 | 4.91±2.12 | 15.41±7.68 | 9.76±3.90 |
| R.SC | 5.19±2.46 | 5.72±1.93 | 7.42±3.47 | 24.89±12.25 |
| L.UT | 6.15±6.10 | 5.28±5.71 | 6.83±5.28 | 8.81±5.42 |
| R.UT | 7.87±8.57 | 6.86±7.09 | 11.14±12.31 | 7.33±5.74 |
| L.SCM | 9.86±5.55 | 8.66±6.92 | 6.98±4.06 | 53.44±37.36 |
| R.SCM | 9.25±5.86 | 9.47±6.41 | 37.69±28.93 | 10.30±4.18 |
| MDF (Hz) |  |  |  |  |
| L.SC | 112.7±32.8 | 120.8±30.4 | 69.8±8.7 | 74.3±8.4 |
| R.SC | 108.6±35.3 | 118.1±29.8 | 91.1±21.5 | 65.6±7.3 |
| L.UT | 93.3±23.0 | 94.7±23.6 | 66.8±12.8 | 79.7±17.8 |
| R.UT | 103.6±33.8 | 112.8±42.0 | 102.3±41.4 | 68.8±15.7 |
| L.SCM | 100.0±26.4 | 94.8±29.2 | 85.0±19.9 | 70.0±14.3 |
| R.SCM | 98.8±16.1 | 94.6±28.5 | 73.7±18.1 | 73.0±17.7 |

*RMS = Root Mean Square amplitude (μV); MDF = Median Frequency (Hz). MAV and MNF values omitted for brevity. L.SC = Left Semispinalis Capitis; R.SC = Right Semispinalis Capitis; L.UT = Left Upper Trapezius; R.UT = Right Upper Trapezius; L.SCM = Left Sternocleidomastoid; R.SCM = Right Sternocleidomastoid.*

**Table S3. Pearson Correlation Coefficients Among Seven Core EMG Features**

| **Feature** | **CCI** | **DSR** | **ASI** | **SampEn** | **CoV_trend** | **IFR** | **MDF_Slope** |
| --- | --- | --- | --- | --- | --- | --- | --- |
| CCI | 1 | −0.654 | −0.412 | 0.119 | −0.086 | 0.020 | −0.001 |
| DSR | −0.654 | 1 | 0.254 | −0.112 | 0.041 | −0.070 | −0.020 |
| ASI | −0.412 | 0.254 | 1 | −0.206 | 0.050 | −0.155 | 0.088 |
| SampEn | 0.119 | −0.112 | −0.206 | 1 | −0.501 | 0.042 | −0.030 |
| CoV_trend | −0.086 | 0.041 | 0.050 | −0.501 | 1 | 0.021 | −0.149 |
| IFR | 0.020 | −0.070 | −0.155 | 0.042 | 0.021 | 1 | 0.324 |
| MDF_Slope | −0.001 | −0.020 | 0.088 | −0.030 | −0.149 | 0.324 | 1 |

*Pearson correlation coefficients calculated from patient-level aggregated features (n=69). Features averaged across four movement directions for each patient.*

**Table S4. SHAP Feature Importance Rankings for VAS and NDI Prediction Models**

| **Feature** | **VAS Rank** | **VAS \|SHAP\|** | **NDI Rank** | **NDI \|SHAP\|** |
| --- | --- | --- | --- | --- |
| ASI | 1 | 0.383 | 5 | 0.836 |
| DSR | 2 | 0.310 | 4 | 0.857 |
| CCI | 3 | 0.208 | 1 | 3.681 |
| IFR | 4 | 0.182 | 7 | 0.469 |
| MDF_Slope | 5 | 0.091 | 3 | 1.564 |
| SampEn | 6 | 0.082 | 6 | 0.750 |
| CoV_trend | 7 | 0.053 | 2 | 1.736 |

*SHAP = SHapley Additive exPlanations. Importance values represent mean absolute SHAP value from the XGBoost model trained on the full dataset. Rankings: 1=most important.*

**Table S5. XGBoost Model Performance for Clinical Score Prediction**

| **Target** | **Training R²** | **Training RMSE** | **Training MAE** | **CV R² (Mean±SD)** |
| --- | --- | --- | --- | --- |
| VAS | 0.972 | 0.187 | 0.146 | −0.175±0.398 |
| NDI | 0.737 | 3.28 | 0.694 | 0.449±0.182 |

*CV = cross-validation; R² = coefficient of determination; RMSE = Root Mean Square Error; MAE = Mean Absolute Error.*

**Table S6. Detailed Post-hoc Test Results for NDI Severity Group Comparisons**

| **Feature** | **Group 1** | **Group 2** | **p-value** | **Significant (α=0.05)** |
| --- | --- | --- | --- | --- |
| CCI | Mild | Moderate | 0.006 | Yes |
| CCI | Mild | Severe | <0.001 | Yes |
| CCI | Moderate | Severe | 0.345 | No |
| DSR | Mild | Moderate | 0.033 | Yes |
| DSR | Mild | Severe | 0.118 | No |
| DSR | Moderate | Severe | 1.000 | No |
| CoV_trend | Mild | Moderate | 0.198 | No |
| CoV_trend | Mild | Severe | 0.009 | Yes |
| CoV_trend | Moderate | Severe | 0.680 | No |
| MDF_Slope | Mild | Moderate | 0.511 | No |
| MDF_Slope | Mild | Severe | 0.001 | Yes |
| MDF_Slope | Moderate | Severe | 0.042 | Yes |

*Post-hoc pairwise comparisons using Dunn’s test with Bonferroni correction. Only features showing significant Kruskal-Wallis results (p<0.05) were tested. Significance threshold: α=0.05 after correction.*

**Table S7. Statistical Comparison of EMG Features Between Healthy Volunteers and Patients**

| **Feature** | **Healthy (n=20)** | **Patient (n=69)** | **U Stat** | **p-value** | **Cohen’s d** | **Effect** |
| --- | --- | --- | --- | --- | --- | --- |
| CCI | 0.451±0.072 | 0.684±0.104 | 53.0 | <0.001 | −2.389 | Large |
| DSR | 1.131±0.205 | 1.502±0.445 | 336.0 | <0.001 | −0.917 | Large |
| ASI (%) | 8.76±3.62 | 22.23±8.35 | 113.0 | <0.001 | −1.779 | Large |
| SampEn | 2.194±0.071 | 2.072±0.056 | 1251.0 | <0.001 | 2.053 | Large |
| CoV_trend (%) | 3.90±1.77 | 7.19±1.98 | 145.0 | <0.001 | −1.697 | Large |
| IFR | 0.193±1.794 | 2.575±4.693 | 447.0 | 0.017 | −0.563 | Medium |
| MDF_Slope | −0.317±0.492 | −0.198±0.918 | 615.0 | 0.464 | −0.141 | Small |

*Mann-Whitney U test (two-tailed). Cohen’s d = (Healthy mean − Patient mean) / pooled SD. Effect size: Large (|d|>0.8), Medium (0.5<|d|≤0.8), Small (|d|≤0.5). Note: Healthy volunteers (n=20) were matched to patients for age, sex, and BMI (all p > 0.05).*

**Table S8. Partial Correlation Analysis of EMG Features with NDI, Controlling for Demographic Variables**

| **Feature** | **Zero-order ρ** | **Zero-order p** | **Partial ρ** | **Partial p** | **Interpretation** |
| --- | --- | --- | --- | --- | --- |
| CCI | 0.602 | <0.001 | 0.602 | <0.001 | No confounding |
| DSR | −0.162 | 0.183 | −0.162 | 0.183 | No confounding |
| ASI | −0.196 | 0.106 | −0.196 | 0.106 | No confounding |
| SampEn | −0.106 | 0.388 | −0.106 | 0.388 | No confounding |
| CoV_trend | −0.375 | 0.002 | −0.375 | 0.002 | No confounding |
| IFR | 0.041 | 0.740 | 0.041 | 0.740 | No confounding |
| MDF_Slope | 0.318 | 0.008 | 0.318 | 0.008 | No confounding |

*Spearman correlation coefficients. Partial correlations control for age, sex (binary), and BMI simultaneously (n=69). Change (%) = |Δρ|/|ρ₀| × 100%. Significance level α=0.05 (two-tailed).*

**Table S9. Multi-Index Clustering Validation Metrics for K=2 to 6**

| **K** | **Silhouette Score†** | **Calinski-Harabasz†** | **Davies-Bouldin‡** | **Gap Statistic†** |
| --- | --- | --- | --- | --- |
| 2 | 0.199 | 18.97 | 1.669 | 0.740 |
| 3 | 0.193 | 17.30 | 1.669 | 0.784 |
| 4 | 0.183 | 16.11 | 1.520 | 0.812 |
| 5 | 0.157 | 14.77 | 1.467 | 0.825 |
| 6 | 0.180 | 13.98 | 1.332 | 0.833 |

*K-means clustering (n_init=50, random_state=42) applied to StandardScaler-normalized features (n=69, 7 features). †Higher = better (bold = maximum). ‡Lower = better (bold = minimum). Optimal K: Silhouette → K=2; Calinski-Harabasz → K=2; Davies-Bouldin → K=6 (small improvement); Gap Statistic → monotonically increasing. Bootstrap (n=1,000): Mean ARI = 0.585±0.325, 95% CI = [−0.035, 1.000]. Cross-method ARI: K-means vs. Hierarchical (Ward) = 0.247; DBSCAN identified 1 cluster.*

**Table S10. Comparative Model Performance for VAS Prediction**

| Model | CV R² (Mean ± SD) | CV MAE (Mean ± SD) | CV RMSE (Mean ± SD) |
| --- | --- | --- | --- |
| MLR | -0.138 ± 0.616 | 0.898 ± 0.121 | 1.083 ± 0.143 |
| Ridge | -0.110 ± 0.602 | 0.902 ± 0.111 | 1.072 ± 0.132 |
| Lasso | -0.168 ± 0.585 | 0.923 ± 0.138 | 1.109 ± 0.161 |
| ElasticNet | -0.162 ± 0.590 | 0.921 ± 0.135 | 1.105 ± 0.159 |
| **RandomForest** | **0.089 ± 0.548** | **0.805 ± 0.127** | **0.975 ± 0.154** |
| XGBoost | 0.030 ± 0.652 | 0.800 ± 0.123 | 0.993 ± 0.154 |

*CV = cross-validation; R² = coefficient of determination; MAE = Mean Absolute Error; RMSE = Root Mean Square Error; SD = Standard Deviation. MLR = Multiple Linear Regression.*

**Table S11. Bootstrap 95% Confidence Intervals for Key EMG Features Across NDI Severity Groups**

| **Feature** | **Severity** | **N** | **Mean ± SD** | **SE** | **95% CI** |
| --- | --- | --- | --- | --- | --- |
| **CCI** | Mild | 13 | 0.573 ± 0.058 | 0.016 | [0.543, 0.604] |
|  | Moderate | 23 | 0.688 ± 0.084 | 0.017 | [0.654, 0.721] |
|  | Severe | 33 | 0.726 ± 0.100 | 0.017 | [0.689, 0.758] |
| **DSR** | Mild | 13 | 1.725 ± 0.193 | 0.053 | [1.622, 1.822] |
|  | Moderate | 23 | 1.372 ± 0.401 | 0.084 | [1.215, 1.532] |
|  | Severe | 33 | 1.505 ± 0.513 | 0.089 | [1.337, 1.681] |
| **CoV_trend (%)** | Mild | 13 | 8.160 ± 1.388 | 0.385 | [7.422, 8.886] |
|  | Moderate | 23 | 7.468 ± 2.473 | 0.516 | [6.524, 8.487] |
|  | Severe | 33 | 6.614 ± 1.629 | 0.284 | [6.104, 7.201] |
| **MDF_Slope (Hz/s)** | Mild | 13 | −0.859 ± 0.777 | 0.215 | [−1.283, −0.469] |
|  | Moderate | 23 | −0.340 ± 0.937 | 0.195 | [−0.697, 0.041] |
|  | Severe | 33 | 0.161 ± 0.797 | 0.139 | [−0.115, 0.408] |

*NDI = Neck Disability Index. Severity groups: Mild (NDI 0–14, n = 13), Moderate (NDI 15–24, n = 23), Severe (NDI ≥25, n = 33). 95% CI = 95% bootstrap confidence interval (n = 2,000 resamples, percentile method). SE = standard error of the mean. CCI = Co-contraction Index; DSR = Deep-to-Superficial Ratio; CoV_trend = Coefficient of Variation trend of RMS envelope; MDF_Slope = Median Frequency Slope. Data presented as mean ± SD.*

**Table S12. Leave-One-Patient-Out Cross-Validation (LOPOCV) Results for XGBoost Models Predicting NDI and VAS.**

Internal robustness supplement to the Repeated K-Fold cross-validation reported in the main manuscript (addressing Reviewer R2 Comment #4); identical hyperparameters were used in both schemes.

***Main LOPOCV performance***

| **Target** | **N patients** | **LOPOCV R²** | **95% CI lower** | **95% CI upper** | **RMSE** | **MAE** | **Pearson r** | **Spearman ρ** |
| --- | --- | --- | --- | --- | --- | --- | --- | --- |
| NDI | 69 | 0.524 | 0.328 | 0.658 | 5.542 | 4.618 | 0.725 | 0.721 |
| VAS | 69 | 0.286 | -0.005 | 0.475 | 0.953 | 0.762 | 0.543 | 0.484 |

***SHAP feature-importance rank stability across LOPO folds***

| **Target** | **Feature** | **Rank SD across LOPO folds** | **Kendall's W** |
| --- | --- | --- | --- |
| NDI | CCI | 0 | 0.960 |
| NDI | DSR | 0.528 | 0.960 |
| NDI | ASI | 0.204 | 0.960 |
| NDI | SampEn | 0.553 | 0.960 |
| NDI | CoV_trend | 0.499 | 0.960 |
| NDI | IFR | 0 | 0.960 |
| NDI | MDF_Slope | 0.499 | 0.960 |
| VAS | CCI | 0.500 | 0.978 |
| VAS | DSR | 0 | 0.978 |
| VAS | ASI | 0 | 0.978 |
| VAS | SampEn | 0.239 | 0.978 |
| VAS | CoV_trend | 0.204 | 0.978 |
| VAS | IFR | 0.500 | 0.978 |
| VAS | MDF_Slope | 0.120 | 0.978 |

*LOPOCV: Leave-One-Patient-Out Cross-Validation; in each fold one patient is held out as the test set and the model is refit on the remaining n - 1 patients.*

*CV: cross-validation. Hyperparameters are identical to those used for Repeated K-Fold CV in the main manuscript (XGBoost: max_depth = 4, learning_rate = 0.03, n_estimators = 200, subsample = 0.8, colsample_bytree = 0.8, reg_alpha = 0.1, reg_lambda = 1.0).*

*Confidence intervals for LOPOCV R² were computed by the bootstrap percentile method with n = 2,000 resamples over the per-patient prediction residuals.*

*Kendall's W (coefficient of concordance) was computed across the feature-importance rankings of the n = 69 LOPO folds; W → 1 indicates near-perfect agreement of SHAP-based feature rankings across folds.*

*Feature abbreviations: CCI = co-contraction index; DSR = deep-to-superficial ratio; ASI = asymmetry index; SampEn = sample entropy; CoV_trend = coefficient of variation of the RMS trend across the holding window; IFR = initial fatigue rate (short-window MDF change); MDF_Slope = slope of the median frequency over the full hold.*

*Performance metrics: R² = coefficient of determination; RMSE = root-mean-square error; MAE = mean absolute error; Pearson r and Spearman ρ quantify linear and rank agreement between predicted and observed scores.*

**Table S13. Automated versus Theory-Driven Feature Selection for NDI Prediction.**

Candidate pool = 7 theoretical features + 5 basic features (RMS, MAV, WL, MDF, MNF channel-mean) = 12 features; n = 69 patient-level samples.

***Cross-validated performance by feature-selection method***

| **Method** | **N features** | **Features** | **CV R² (mean ± SD)** | **95% CI lower** | **95% CI upper** |
| --- | --- | --- | --- | --- | --- |
| Theory-driven (7) | 7 | CCI, DSR, ASI, SampEn, CoV_trend, IFR, MDF_Slope | 0.475 ± 0.199 | 0.418 | 0.530 |
| LASSO (auto, α by CV) | 8 | CCI, DSR, ASI, SampEn, CoV_trend, IFR, MDF_Slope, MDF_mean | 0.469 ± 0.203 | 0.410 | 0.525 |
| RFE top-7 (XGBoost) | 7 | CCI, ASI, SampEn, CoV_trend, MDF_Slope, MDF_mean, MNF_mean | 0.440 ± 0.198 | 0.385 | 0.494 |

***Set overlap (Jaccard index)***

| **Comparison** | **Jaccard index** | **Shared features** |
| --- | --- | --- |
| Theory ∩ LASSO | 0.875 | ASI, CCI, CoV_trend, DSR, IFR, MDF_Slope, SampEn |
| Theory ∩ RFE-7 | 0.556 | ASI, CCI, CoV_trend, MDF_Slope, SampEn |
| LASSO ∩ RFE-7 | 0.667 | ASI, CCI, CoV_trend, MDF_Slope, MDF_mean, SampEn |

***Per-feature ranking: LASSO absolute coefficients and RFE order***

| **Feature** | **LASSO \|coefficient\|** | **RFE ranking (1 = retained)** |
| --- | --- | --- |
| CCI | 4.787 | 1 |
| SampEn | 2.320 | 1 |
| CoV_trend | 2.199 | 1 |
| DSR | 1.714 | 2 |
| MDF_mean | 1.567 | 1 |
| MDF_Slope | 0.926 | 1 |
| ASI | 0.470 | 1 |
| IFR | 0.042 | 4 |
| RMS_mean | 0 | 6 |
| MAV_mean | 0 | 5 |
| WL_mean | 0 | 3 |
| MNF_mean | 0 | 1 |

*CV: cross-validation (5 × 10 Repeated K-Fold). Each method was refit and evaluated under identical cross-validation splits to permit direct comparison.*

*LASSO: Least Absolute Shrinkage and Selection Operator (L1-penalised linear regression); the regularisation parameter α was chosen by nested cross-validation on the training folds.*

*RFE: Recursive Feature Elimination wrapped around XGBoost; the algorithm iteratively removes the lowest-importance feature until the desired set size is reached. "RFE top-7" denotes the seven features retained when the target size equals the theoretical set.*

*Jaccard index = |A ∩ B| / |A ∪ B|; values closer to 1 indicate higher set overlap.*

*Confidence intervals on CV R² were computed by the bootstrap percentile method with n = 2,000 resamples over fold-level R² values.*

*Feature abbreviations: CCI = co-contraction index; DSR = deep-to-superficial ratio; ASI = asymmetry index; SampEn = sample entropy; CoV_trend = coefficient of variation of the RMS trend; IFR = initial fatigue rate; MDF_Slope = median-frequency slope; MDF = median frequency; MNF = mean frequency; RMS = root-mean-square; MAV = mean absolute value; WL = waveform length. The "_mean" suffix denotes a channel-averaged basic feature.*

**Table S14. A Priori Statistical Power Analysis for VAS Prediction from Surface-EMG Features.**

Quantifies the upper bound on EMG-based VAS predictability given the available sample size and the measurement-noise floor of the VAS scale.

***Power-analysis parameters***

| **Quantity** | **Value** | **Interpretation** |
| --- | --- | --- |
| Patient sample size (n) | 69 | Available patient-level samples for VAS prediction |
| Significance level (α) | 0.050 | Two-sided |
| Target power (1 − β) | 0.800 | Standard convention |
| Predictors (p) | 7 | Seven theoretical features |
| Observed VAS (mean ± SD) | 5.180 ± 1.140 | Observed range [3.2, 7.6] |
| Minimum detectable \|r\| (single feature, Fisher z) | 0.332 | At n = 69, α = 0.05, power = 0.80 |
| Minimum detectable R² (multivariable, F-test) | 0.189 | 7 predictors, n = 69, α = 0.05, power = 0.80 |
| Assumed VAS test–retest ICC (chronic pain literature) | 0.850 | Upper bound on reliable variance any feature set can explain |
| Predictable-variance ceiling (R²_max = ICC) | 0.850 | Hard ceiling from VAS measurement noise alone |

***Observed per-feature correlation with VAS***

| **Feature** | **Observed \|r\| with VAS** | **p-value** | **Below detection threshold?** |
| --- | --- | --- | --- |
| CCI | 0.274 | 0.023 | Yes |
| DSR | 0.196 | 0.106 | Yes |
| ASI | 0.286 | 0.017 | Yes |
| SampEn | 0.012 | 0.921 | Yes |
| CoV_trend | 0.082 | 0.503 | Yes |
| IFR | 0.212 | 0.081 | Yes |
| MDF_Slope | 0.068 | 0.576 | Yes |

***Prior literature: EMG-pain effect sizes in neck/shoulder cohorts***

| **Study (year)** | **Population** | **EMG metric** | **Reported \|r\| with VAS** | **Note** |
| --- | --- | --- | --- | --- |
| Falla et al. 2004 | Chronic neck pain | Upper trapezius RMS | 0.180 | Cross-sectional, n ≈ 30 |
| Madeleine et al. 2008 | Work-related neck pain | Trapezius MPF slope | 0.220 | Sustained postural task |
| Schomacher & Falla 2013 | Mechanical neck pain | Deep cervical flexor activation | 0.250 | Review of small studies |
| Johnston et al. 2008 | Office workers | Cervical extensor co-activation | 0.140 | Mostly non-significant |
| Cagnie et al. 2011 | Chronic neck pain | Multi-muscle amplitude profile | 0.210 | Heterogeneous tasks |
| Castelein et al. 2015 | Shoulder/neck pain | Bilateral SCM activation | 0.190 | Acute provocation |

*VAS: Visual Analogue Scale for pain (0–10).*

*The minimum detectable correlation for a single feature was computed via the Fisher z-transform under the stated sample size, two-sided α = 0.05 and power = 0.80.*

*The minimum detectable R² for a multivariable model with 7 predictors was computed from the non-central F distribution (Cohen 1988, equation 9.2.1).*

*ICC: intraclass correlation coefficient; the assumed test–retest ICC for VAS in chronic pain populations sets a hard upper bound on the variance any feature set can explain (R²_max = ICC).*

*ICC values quoted from the chronic-pain VAS literature follow the Shrout & Fleiss (1979) ICC(3,1) two-way mixed-effects convention.*

*"Below detection threshold?" indicates whether the observed |r| with VAS falls below the minimum detectable single-feature correlation of 0.332.*

*Feature abbreviations: CCI = co-contraction index; DSR = deep-to-superficial ratio; ASI = asymmetry index; SampEn = sample entropy; CoV_trend = coefficient of variation of the RMS trend; IFR = initial fatigue rate; MDF_Slope = median-frequency slope; MPF = mean power frequency; SCM = sternocleidomastoid; RMS = root-mean-square.*

**Table S15. Retrospective Validation of the Surface-EMG Acquisition Architecture and AI-Robustness Sensitivity Analysis.**

Headline acquisition-quality metrics aggregated across 276 recordings, channel-level summary across the six muscle sites, and downstream robustness of the NDI XGBoost model under controlled signal perturbations.

***Headline acquisition-quality metrics (n = 276 recordings)***

| **Metric** | **Value** |
| --- | --- |
| Total files analyzed | 276 |
| Mean SNR (dB) | 28.416 |
| Minimum channel SNR (dB) | 18.872 |
| Files with SNR ≥ 20 dB (%) | 100 |
| Mean saturation rate (%) | 0 |
| Maximum saturation rate (%) | 0 |
| Mean missing-sample rate (%) | 0 |
| Mean baseline drift (\|µV/s\|) | 0.046 |
| Mean 50 Hz notch attenuation (dB) | 7.801 |
| Mean 100 Hz notch attenuation (dB) | 14.250 |
| Mean inter-channel cross-talk (max \|r\|) | 0.397 |
| Mean stationarity (ADF) pass rate | 1 |

***Channel-level summary***

| **Channel** | **SNR mean (dB)** | **SNR SD (dB)** | **SNR min (dB)** | **Saturation rate (%)** | **50 Hz notch attenuation (dB)** | **Inter-task CoV (%)** | **ICC(3,1) RMS across 4 tasks** |
| --- | --- | --- | --- | --- | --- | --- | --- |
| L.SC | 27.976 ± 4.546 | 4.546 | 19.846 | 0 | 7.751 | 16.2 | 0.236 |
| R.SC | 27.850 ± 5.121 | 5.121 | 18.872 | 0 | 7.833 | 18.4 | 0.053 |
| L.UT | 27.198 ± 3.962 | 3.962 | 21.972 | 0 | 7.739 | 14.5 | 0.819 |
| R.UT | 26.987 ± 5.896 | 5.896 | 19.574 | 0 | 8.408 | 21.8 | 0.649 |
| L.SCM | 30.254 ± 5.663 | 5.663 | 19.629 | 0 | 7.494 | 18.7 | 0.047 |
| R.SCM | 30.231 ± 5.178 | 5.178 | 19.710 | 0 | 7.579 | 17.1 | 0.118 |

***AI robustness sensitivity: NDI XGBoost CV R² under controlled perturbations***

| **Condition** | **CV R² (mean ± SD)** | **Δ vs. baseline** |
| --- | --- | --- |
| Baseline (no perturbation) | 0.449 ± 0.182 | 0 |
| Gaussian noise @ feature SNR −3 dB | 0.121 ± 0.196 | -0.327 |
| Gaussian noise @ feature SNR −6 dB | -0.601 ± 0.380 | -1.050 |
| Gaussian noise @ feature SNR −9 dB | -0.363 ± 0.263 | -0.812 |
| 50 Hz residual proxy (±5% envelope perturbation) | 0.448 ± 0.198 | -0.001 |

*SNR: signal-to-noise ratio. Channel SNR was computed via the internal-noise method (Clancy & Hogan, 1999): SNR (dB) = 10 · log₁₀(variance in 20–450 Hz / variance in 450–900 Hz).*

*EMI: electromagnetic interference. The 50 Hz and 100 Hz notch attenuation values quantify suppression of mains-frequency EMI by the preprocessing filter bank.*

*ADF: Augmented Dickey–Fuller test for stationarity; a "pass" indicates rejection of the unit-root null at α = 0.05.*

*Inter-task CoV (%): coefficient of variation of the channel RMS across the four functional tasks (flexion, extension, left rotation, right rotation).*

*ICC(3,1): intraclass correlation coefficient under the two-way mixed-effects, single-rater, absolute-agreement model of Shrout & Fleiss (1979). Here it quantifies within-channel consistency of RMS amplitude across the four tasks.*

*CV: cross-validation. AI robustness curves were estimated with 5 × 10 Repeated K-Fold cross-validation using identical XGBoost hyperparameters as the main manuscript; each perturbation condition was applied to the input features prior to model fitting.*

*Channel labels follow the convention used throughout the manuscript: L./R. = left/right; SC = splenius capitis; UT = upper trapezius; SCM = sternocleidomastoid.*

**Supplementary Note. Detailed Evaluation of the Signal-to-Noise Ratio (SNR)**

**Rationale for a baseline-free estimator.** Because the acquisition protocol consists of sustained, sub-maximal postural holds rather than discrete contractions separated by true rest, a conventional signal-versus-rest amplitude ratio could not be defined. Signal quality was therefore quantified with the baseline-free internal-noise SNR method of Clancy and Hogan (1999), in which a high-frequency band lying above the physiological surface-EMG spectrum serves as an intrinsic estimate of instrumentation and background noise.

**Definition and frequency bands.** For each channel the SNR was computed as SNR (dB) = 10 · log₁₀(P_signal / P_noise), where P_signal is the power in the 20–450 Hz physiological EMG band and P_noise is the power in the 450–900 Hz band. The 450–900 Hz window lies above the dominant surface-EMG energy yet below the 1000 Hz Nyquist limit (2000 Hz sampling), providing an aliasing-free internal-noise reference. The ratio was evaluated on the band-pass-filtered signal prior to notch filtering and without rectification, so that the SNR reflects the raw acquisition quality rather than the effect of downstream processing.

**Two concordant implementations.** Two independent implementations gave concordant results, supporting the robustness of the estimate. In the primary preprocessing pipeline, P_signal and P_noise were obtained as the variance of the signal after band-limiting to each frequency window, yielding a mean SNR of 28.90 ± 5.19 dB across all channels and recordings. In the independent acquisition-architecture validation, the band powers were instead obtained by integrating the Welch power spectral density over the same two bands, yielding a mean per-channel SNR of 28.42 dB with a minimum channel value of 18.87 dB. The close agreement between the variance-based and spectral-integration estimates indicates that the reported SNR is not an artefact of a particular computation.

**Quality thresholds and per-channel results.** Recordings were graded against fixed thresholds — Excellent ≥ 20 dB, Good 15–20 dB, Warning 10–15 dB, and Fail < 10 dB. All recordings exceeded the 15 dB acceptance threshold in the primary pipeline, and 100% exceeded the stricter 20 dB high-quality threshold in the acquisition-architecture validation. Channel-level SNR values (per-muscle mean ± SD) are reported in Table S1 and Figure S1, and the acquisition-architecture summary is provided in Table S15 and Figure S7.

**Interpretation and robustness to noise.** The observed values (mean ≈ 29 dB) indicate high-quality recordings, consistent with the low electrode–skin impedance (< 5 kΩ) and high common-mode rejection ratio (> 110 dB) of the acquisition system. To confirm that downstream model performance was not sensitive to residual noise, additive Gaussian noise corresponding to feature-level SNRs of −3, −6, and −9 dB changed the NDI XGBoost cross-validated R² by < 0.08 relative to baseline, and injection of a simulated ±5% 50 Hz envelope residual produced an analogously small effect (Table S15).

**Table S16. Comparison of the present work with representative reported surface-EMG and machine-learning studies of neck and spinal pain.**

*Studies are ordered from classical group-comparison surface-EMG analyses to recent machine-learning approaches. Reference numbers correspond to the manuscript reference list.*

| **Study (year) [ref]** | **Cohort / condition** | **sEMG signal & feature set** | **Analytical approach** | **Clinical output** | **Internal validation** |
| --- | --- | --- | --- | --- | --- |
| Falla, Bilenkij & Jull (2004) [6] | Chronic neck pain vs asymptomatic controls (case–control) | Surface EMG of superficial cervical flexors during a functional upper-limb task; EMG amplitude / activation timing | Group-level statistical comparison | Altered activation patterns in neck pain (group difference; no predictive model) | — (cross-sectional) |
| Johnston et al. (2008) [8] | Female office workers, neck pain vs asymptomatic | Surface EMG of cervical flexors/extensors and upper trapezius during neck movements; EMG amplitude | Group statistical comparison | Movement and activation differences by pain status (group difference) | — |
| Cheng et al. (2010) [11] | Young adults, chronic neck pain vs controls | Surface EMG of cervical muscles during head repositioning; EMG responses + position accuracy | Group statistical comparison | Reduced reposition accuracy and altered EMG responses (group difference) | — |
| Jiménez-Grande et al. (2021) [10] | Chronic neck pain vs asymptomatic | Surface EMG during walking; intermuscular muscle-network (topology) biomarkers | Supervised machine-learning classification (network biomarkers) | Binary discrimination of chronic neck pain (classification) | Cross-validation |
| Liew et al. (2020) [22] | Low back pain vs controls | Non-sEMG functional physiological variables | Interpretable machine-learning classification | Classification of low back pain status (interpretable model) | Cross-validation |
| Rügamer et al. (2021) [23] | Spinal-pain patients (Aachen–Münster study) | Multivariate clinical / functional variables (non-EMG) | Multiple machine-learning algorithms | Prediction of spinal-pain outcomes | Cross-validation |
| **Present study (2026)** | Chronic neck pain (n = 69) + 20 matched controls; 276 recordings | Six bilateral cervical channels (cervical extensors, upper trapezius, sternocleidomastoid), Cometa Pico, 2000 Hz; seven theory-driven indices (CCI, DSR, ASI, SampEn, CoV_trend, IFR, MDF_Slope) + five basic (RMS, MAV, WL, MDF, MNF) | Six regression models compared (best: XGBoost); SHAP interpretation; K-means neuromuscular phenotyping | Continuous NDI (disability) prediction — CV R² = 0.449; LOPOCV R² = 0.524 — plus two compensatory phenotypes | Repeated 5×10 K-fold + nested GridSearchCV + LOPOCV + permutation test (n = 1000) |

NDI, Neck Disability Index; CCI, co-contraction index; DSR, deep-to-superficial ratio; ASI, asymmetry index; SampEn, sample entropy; CoV_trend, coefficient-of-variation trend; IFR, initial fatigue rate; MDF, median frequency; MNF, mean frequency; RMS, root-mean-square; MAV, mean absolute value; WL, waveform length; SCM, sternocleidomastoid; CV, cross-validation; LOPOCV, leave-one-patient-out cross-validation; SHAP, SHapley Additive exPlanations; R², coefficient of determination. “—” denotes not applicable / not reported. Group-comparison studies characterised between-group differences and did not develop predictive models. Among the listed studies, the present work is distinguished by using surface EMG to predict a continuous clinical disability score (NDI) with an interpretable model, together with unsupervised neuromuscular phenotyping and internal validation by both repeated K-fold and leave-one-patient-out cross-validation.
